# Origins Left, Right, and Centre: Increasing the Number of Initiation Sites in the *Escherichia coli* Chromosome

**DOI:** 10.3390/genes9080376

**Published:** 2018-07-27

**Authors:** Juachi U. Dimude, Monja Stein, Ewa E. Andrzejewska, Mohammad S. Khalifa, Alexandra Gajdosova, Renata Retkute, Ole Skovgaard, Christian J. Rudolph

**Affiliations:** 1 Division of Biosciences, College of Health and Life Sciences, Brunel University London, Uxbridge UB8 3PH, UK; Juachi.Dimude@brunel.ac.uk (J.U.D.); Monja.St@gmx.com (M.S.); ewa.andrzejewska95@gmail.com (E.E.A.); 1443069@brunel.ac.uk (M.S.K.); 1618328@brunel.ac.uk (A.G.); 2 School of Life Sciences, University of Warwick, Gibbet Hill Campus, Coventry CV4 7AL, UK; R.Retkute@warwick.ac.uk; 3 Department of Science and Environment, Roskilde University, DK-4000 Roskilde, Denmark; olesk@ruc.dk

**Keywords:** DNA replication, replication termination, *ter*/Tus complex, replication-transcription conflicts, chromosome dynamics, bacterial chromosome structure

## Abstract

The bacterium *Escherichia coli* contains a single circular chromosome with a defined architecture. DNA replication initiates at a single origin called *oriC.* Two replication forks are assembled and proceed in opposite directions until they fuse in a specialised zone opposite the origin. This termination area is flanked by polar replication fork pause sites that allow forks to enter, but not to leave. Thus, the chromosome is divided into two replichores, each replicated by a single replication fork. Recently, we analysed the replication parameters in *E. coli* cells, in which an ectopic origin termed *oriZ* was integrated in the right-hand replichore. Two major obstacles to replication were identified: (1) head-on replication–transcription conflicts at highly transcribed *rrn* operons, and (2) the replication fork trap. Here, we describe replication parameters in cells with ectopic origins, termed *oriX* and *oriY*, integrated into the left-hand replichore, and a triple origin construct with *oriX* integrated in the left-hand and *oriZ* in the right-hand replichore. Our data again highlight both replication–transcription conflicts and the replication fork trap as important obstacles to DNA replication, and we describe a number of spontaneous large genomic rearrangements which successfully alleviate some of the problems arising from having an additional origin in an ectopic location. However, our data reveal additional factors that impact efficient chromosome duplication, highlighting the complexity of chromosomal architecture.

## 1. Introduction

**Figure 1:**
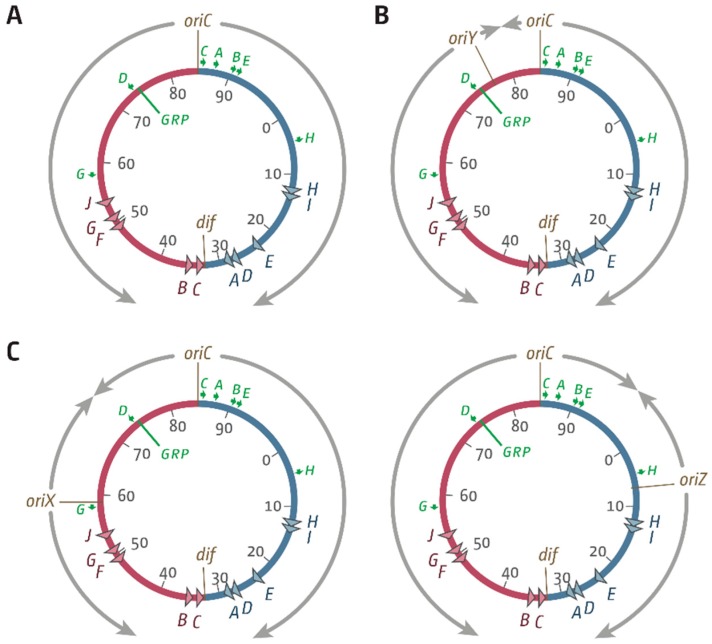
Schematic representation of the replichore arrangement of *Escherichia coli* chromosomes with ectopic replication origins in different locations. (**A**) Normal replichore arrangement in *E. coli*. The origin, *oriC*, and the *dif* chromosome dimer resolution site are indicated. *ter* sites are shown by triangles and are identified by their corresponding letter (“A” indicates the *terA* site). The numbers represent the minutes of the standard genetic map (0–100 min). Green arrows represent location and direction of transcription of the seven *rrn* operons: A–E, G, and H. The location marked “GRP” indicates a tight cluster of genes coding for ribosomal proteins, all of which are transcribed co-directionally with replication coming from *oriC*. (**B**) Integration site of a 5 kb *oriC* fragment termed *oriY* into *malT* upstream of the *rrnD* operon. (**C**) Integration sites of 5 kb *oriC* fragments into *pheA* upstream of the *rrnG* operon, termed *oriX* (this study), and near the *lacZYA* operon, termed *oriZ* [18,19].

The ability to accurately duplicate the genetic material and faithfully transmit it to daughter cells is a fundamental necessity of life. An important regulatory step for the initiation of the DNA duplication process in all organisms is the assembly of fully functional replisomes at defined origin sequences [1,2]. While eukaryotic cells replicate their genomes from hundreds or thousands of origins [1], the number of initiation sites in bacteria is mostly restricted to a single origin per chromosome (*oriC*) [3,4]. In *Escherichia coli*, initiation of DNA replication at *oriC* is tightly controlled by the main initiator protein DnaA, which facilitates recruitment of two replisomes [2,5,6,7]. These replisomes proceed in opposite directions around the circular chromosome with very high speed and accuracy until they eventually fuse within a specialised termination area opposite the origin (Figure 1A) [8,9]. The area is flanked by 10 primary *ter* sequences *A–J*. If bound by Tus protein, these *ter* sites form polar traps that allow forks to enter, but not to leave [8,10,11]. The *E. coli* chromosome is thereby divided into two replichores, each being replicated by a single replication fork [8,10,11,12].

Bacteria can tolerate the integration of a second replication origin or movement of the origin into an ectopic location, but both scenarios cause serious problems. Movement of *oriC* in *Bacillus subtilis* to an ectopic location revealed that forks replicating the chromosome in an orientation opposite to normal were significantly slowed at highly transcribed regions, such as the *rrn* operons [13,14], supporting the idea that head-on collisions between replication and transcription are problematic for ongoing DNA replication [15,16]. Introduction of a second replication origin also appears to be difficult to tolerate. Integration of an inducible plasmid origin ~450 kb away from *oriC* was shown to be active, but repressed firing of *oriC* [17].

In a recent study, Wang and colleagues reported the integration of a 5 kb *oriC* fragment called *oriZ* near the *lac* operon at 7.4 min into the *E. coli* chromosome, halfway into the right-hand replichore (Figure 1C) [19]. *oriC^+^ oriZ^+^* cells grew with doubling times similar to wild-type cells, and cell biological observations confirmed that both origins fire simultaneously [19]. The authors also observed that in *ΔoriC oriZ^+^* cells, in which the chromosome is replicated exclusively from the ectopic origin, the doubling time is only marginally longer than in wild-type cells [19], much in contrast to the studies in *B. subtilis* [13,14]. When we regenerated the relevant strains to study their replication dynamics, we found that the doubling time of *ΔoriC oriZ^+^* cells was increased from 20 to over 40 min, demonstrating that cells seriously struggle to grow. The replication profiles of these strains revealed two major obstacles to replication. Firstly, the ectopic *oriZ* disrupts the normal replichore arrangement, with the clockwise replication fork reaching the termination area much quicker than the counter-clockwise fork coming from *oriC*. Consequently, the vast majority of forks are blocked by the replication fork trap. Secondly, replication initiated at *oriZ* and traversing the chromosome opposite to normal is also significantly inhibited by the highly transcribed *rrn* operons *rrnH* and *rrnCABE*, all of which are transcribed co-directionally with replication coming from *oriC* [18], in line with the results in *B. subtilis* [13,14]. Our data show that the slow growth of *ΔoriC oriZ^+^* cells can be partially suppressed by (a) inactivation of the replication fork trap by deletion of *tus* and (b) an rpoB*35 point mutation, which reduces the stability of RNA polymerase–DNA complexes, thereby alleviating conflicts between replication and transcription [18]. However, when we investigated why the original *ΔoriC oriZ^+^* construct by Wang and colleagues [19] was growing so quickly, we found a different suppressor mutation altogether: the strain carried a gross chromosomal rearrangement that inverted almost the entire portion of the chromosome that would otherwise have been replicated in the wrong orientation from *oriZ,* including the *rrnCABE* operon cluster, thereby realigning replication and transcription [18].

This study describes attempts to integrate ectopic replication origins at two defined locations into the opposite, left-hand replichore. In contrast to *rrn* operons *CABE* and *H* in the right-hand replichore, the left-hand replichore only contains *rrn* operons *D* and *G*, as well as a cluster of genes encoding for ribosomal proteins (Figure 1). We therefore hypothesised that integration of an ectopic origin into the left-hand replichore might be less problematic. However, the results presented suggest the opposite. Integration of an active 5 kb origin fragment, termed *oriY*, upstream of *rrnD* was not possible. Given that no *rrn* operons would be encountered head-on by replication starting from this location, the inability to integrate a functional origin in this location suggests that multiple factors must contribute towards origin activity. Integration of a functional 5 kb origin fragment, termed *oriX*, just upstream of *rrnG* into the left-hand replichore was successful, but *ΔoriC oriX^+^* cells grew even more slowly than *ΔoriC oriZ^+^* cells and rapidly accumulated suppressor mutations, some of which are characterised. Finally, we report the successful construction of *oriC^+^ oriX^+^ oriZ^+^* cells. In this triple-origin background, all origins are active in principle, but both ectopic origins show a reduced activity relative to *oriC*. Our results reiterate that both the termination area and head-on replication-transcription encounters act as severe obstacles for chromosomal replication if the replichore arrangement is asymmetric. However, our inability to integrate a functional *oriY,* the slow growth of *ΔoriC oriX^+^* cells, and the preference for *oriC* in triple-origin cells strongly support the idea that a number of different factors influence origin activity and successful genome duplication in the presence of additional ectopic replication initiation sites.

## 2. Material and Methods

### 2.1. Bacterial Strains and General Methods

For *E. coli* K12 strains, see Table 1. Strains were constructed via P1*vir* transductions [20] or by single-step gene disruptions [21].

### 2.2. Growth Media

Luria broth (LB) and agar was modified from Luria and Burrous [27] as follows: 1% tryptone (Bacto™, BD Biosciences, Franklin Lakes, NJ, USA), 0.5% yeast extract (Bacto™, BD Biosciences), and 0.05% NaCl (Sigma Aldrich, St. Louis, MO, USA). The pH was adjusted to 7.4. M9 minimal medium (Bacto™, BD Biosciences) containing 15 g/L KH_2_PO_4_, 64 g/L Na_2_HPO_4_, 2.5 g/L NaCl, and 5.0 g/L NH_4_Cl. Before use, MgSO_4_, CaCl_2_, and glucose were added from sterile-filtered stock solutions to final concentrations of 2 mM, 0.1 mM, and 0.2%, respectively, according to the manufacturer’s recommendation. Doubling times of MG1655 in our growth media were 19.3 ± 1.7 min in LB and 68.8 ± 6.2 min in M9 glucose.

### 2.3. Marker Frequency Analysis by Deep Sequencing

Marker frequency analysis by deep sequencing was performed as described before [18]. See the Supplementary Methods section for a detailed description. All relevant raw sequencing data can be accessed at the European Nucleotide Archive (http://www.ebi.ac.uk/ena/data/view/PRJEB9476).

### 2.4. Locally Estimated Scatterplot Smoothing (LOESS) Regression

A Locally Estimated Scatterplot Smoothing (LOESS) regression allows for a simplified visualisation of complex data sets. For a LOESS regression, relatively simple models are fitted to defined small subsets of data points in order to develop a function describing the deterministic part of the variation in the data. Weighted least-squares are used to fit a low-degree polynomial to a specified percentage of data points. Data points are weighted by a smooth decreasing function of their distance to the smoothed point, giving more weight to points closer to the point whose response is being estimated, while less weight is given to points further away. We used a second-order polynomial for local fit, tricube as weight function, and set a fraction of data used for smoothing to 10%, which corresponds to a smoothing window around 460 kbp [28]. To account for circularity of the chromosome, periodic boundary conditions were used.

### 2.5. Growth Curves

Samples from cultures of a strain grown over night in LB broth were diluted 100-fold in fresh broth and incubated with vigorous aeration at 37 °C until A_600_ reached 0.48. The only exceptions were *ΔoriC oriX^+^* backgrounds, for which growth was initiated from a single colony from a streak plate to avoid suppressors formed in the overnight culture outgrowing the slow-growing *ΔoriC oriX^+^* cells. Upon reaching an A_600_ of 0.48, the culture was diluted 100-fold in prewarmed fresh broth and grown under identical conditions. Samples were taken every 30 min, diluted to 10^−7^ in M9 minimal medium without added glucose, and 10 μL aliquots of each dilution were dropped onto LB agar plates. For each dilution series, two sets of drops were spotted. Colonies were counted after incubation for 18–24 h at 37 °C. Mean colony numbers from both spots were calculated and a growth curve plotted. A suitable period where growth was exponential was selected (usually between 60 and 180 min following dilution into fresh LB). For calculation of the doubling time, the LINEST function in Microsoft Excel 2016 (Microsoft, Redmont, WA, USA) was used to determine linear regression parameters for data points, which were calculated from averages per time point of between three and eight independent experiments. The doubling times of strains shown in Table 2 and Table 3 were carried out in sets. Thus, relevant controls, such as MG1655, *oriC^+^ oriZ^+^*, and *oriC^+^ oriX^+^*, were always measured in parallel to the strains of interest, explaining the slight variations of the doubling times of these strains in the respective tables. Doing so allowed us to largely avoid the comparison of doubling times generated under potentially slightly varying conditions.

### 2.6. Mathematical Modelling

See Supplementary Methods for a detailed description of the mathematical modelling.

## 3. Results

### 3.1. Ectopic Replication Origins in the Left-Hand Replichore

Previously, we investigated replication parameters in strains in which a 5 kb *oriC* fragment called *oriZ* was integrated about 1 Mbp away from the native *oriC* in the right-hand replichore [18,19]. Here, we attempted to integrate another copy of the 5 kb *oriC* fragment into two separate locations within the left-hand replichore. Our previous study had identified *rrn* operons *C*, *A*, *B*, *E*, and *H* as major obstacles to the progression of replication forks coming from the ectopic origin [18]. We speculated that the opposite replichore might pose fewer problems, as only two, rather than five, *rrn* operons are present (Figure 1). We attempted to integrate one 5 kb *oriC* fragment called *oriY* into the *malT* gene at 76.5 min, which is upstream of *rrnD*. The location allows forks coming from *oriY* to progress without any *rrn* operons in their way (Figure 1B). A second construct termed *oriX* was integrated into *pheA* at 59 min, an integration location that is roughly equivalent to the *oriZ* location in terms of replichore length (Figure 1C). The *pheA* gene is just upstream of *rrnG*. Thus, only *rrn* operon *D* and a cluster of genes coding for ribosomal proteins will be encountered in a direction opposite to normal in *ΔoriC oriX^+^* cells (Figure 1).

**Figure 2:**
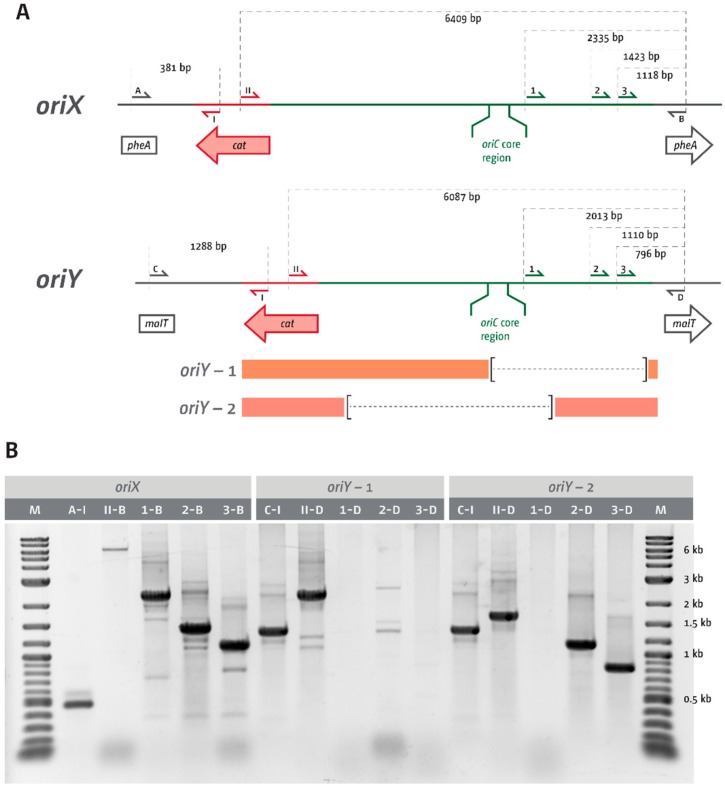
PCR confirmation of *oriX* and *oriY* integration cassettes into the chromosome. (**A**) Schematic representation of the integration region following successful integration of *oriX* into *pheA* or *oriY* into *malT*, respectively. Primers are identified according to their position with letters, numbers, or roman numerals. Primer binding sites are indicated. The orange bars below the *oriY* scheme indicate the likely regions where truncation has taken place, taking into consideration the overall length of the integrated region, as well as the presence and absence of defined primer binding sites, as shown in (**B**). The dashed lines represent the approximate sizes of truncations. (**B**) Agarose gel electrophoresis of PCRs with primers highlighted in (**A**) on templates in which either *oriX* or *oriY* is integrated into the chromosome. Sizes of relevant marker fragments (2-log kb ladder, NEB) are indicated. The primer combinations used for the individual PCRs are given directly above the relevant lane (primers A and I shown in (**A**) are given as A-I). An inverted gel image is shown for clarity.

Both chromosomal integrations resulted in colonies with the correct antibiotic resistance. However, deletion of *oriC* was only possible in *oriC^+^ oriX^+^* cells; we failed to generate a *ΔoriC oriY^+^* construct. PCR verification of two of the *oriY* constructs demonstrated one partial truncation and one complete loss of the *oriC* core elements (Figure 2), explaining the lack of functionality. A repeat of the chromosomal integration directly into MG1655 again did not result in constructs with a functional *oriY*. We do not currently know what is causing the inability to integrate *oriY* into the chromosome, given *oriX*, which was amplified from the same template, could be integrated without difficulty.

### 3.2. oriX Is Active in Double-Origin Cells

**Figure 3:**
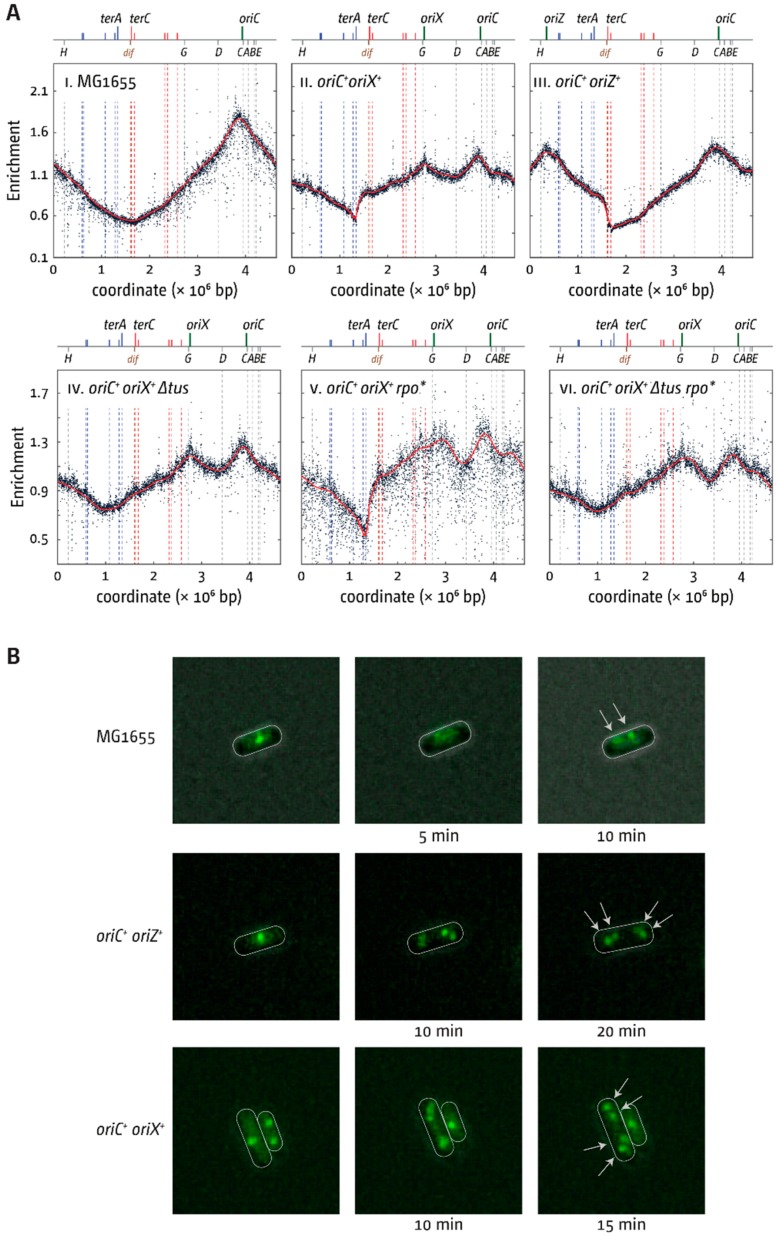
Replication dynamics in *E. coli* cells with one and two replication origins. (**A**) Marker frequency analysis (MFA) of *E. coli oriC^+^*, *oriC^+^ oriX^+^*, and *oriC^+^ oriZ^+^* cells, and impact of *∆tus* and an rpo* point mutation on these cells. The number of reads (normalised against reads for a stationary phase wild-type control) is plotted against the chromosomal location. A schematic representation of the *E. coli* chromosome showing positions of *oriC* and *oriX* (green line) and *ter* sites (above), as well as *dif* and *rrn* operons A–E, G, and H (below), is shown above the plotted data. The strains used were MG1655 (*oriC^+^*), RCe504 (*oriC^+^ oriZ^+^*), JD1181 (*oriC^+^ oriX^+^*), JD1203 (*oriC^+^ oriX^+^ ∆tus*), JD1190 (oriC^+^ oriX^+^ rpo*), and JD1205 (oriC^+^ oriX^+^ ∆tus rpo*). (**B**) Visualisation of replisomes (Ypet-DnaN) in wild-type, *oriC^+^ oriX^+^*, and *oriC^+^ oriZ^+^* cells. Cells were grown in M9 minimal salts medium with 0.2% glucose and transferred onto a thin agarose pad of the same medium on a microscopy slide (see Material and Methods). Slides were transferred into a chamber heated to 37 °C and fluorescent foci in single cells tracked over time. The strains used were AS1062 (*ypet-dnaN*), RCe749 (*oriC^+^ oriZ^+^ ypet-dnaN*), and RCe751 (*oriC^+^ oriX^+^ ypet-dnaN*).

Marker frequency analysis (MFA) was used to investigate the replication profile of *oriC^+^ oriX^+^* cells (Figure 3A). Given that all replication profiles of our previous *oriZ* study were generated from cultures grown in LB broth [18], all samples were grown under similar conditions to enable a direct comparison. The replication profile of *oriC^+^ oriX^+^* cells showed similar features to the previously obtained *oriC^+^ oriZ^+^* profile (Figure 3A). The MFA confirmed that *oriX* was active (Figure 3(AII)).

There appears to be a minor difference in peak height between *oriC* and *oriX.* Our subsequent analysis has shown that this is caused by the column purification procedure to extract genomic DNA (gDNA) for deep sequencing. Insufficient proteolytic digest causes DNA fragments to be lost in areas where protein–DNA complexes are particularly tight or frequent, such as *rrn* operons or *ter*/Tus complexes, as proteins still bound to DNA fragments are eluted from the DNA-binding column (see Supplementary Methods and Figure S1). For *oriX*, it is the proximity of *rrnG* that causes a mild under-representation of the region, which results in a reduced peak height. After identifying this issue, resequencing of an *oriC^+^ oriX^+^* construct following phenol–chloroform extraction of gDNA demonstrated that both *oriC* and *oriX* are active at similar frequencies (Figure S2).

To confirm that both origins are simultaneously active, a strain was used in which the bright YFP derivative YPet was fused to the N-terminus of the β-sliding clamp, encoded by the *dnaN* gene, as reported [19]. To avoid the complexity of overlapping rounds of DNA replication, cells were grown in M9 minimal medium with 0.2% glucose (called M9 hereafter; see Material and Methods). Time-lapse microscopy of otherwise wild-type cells showed that, under these conditions, replisomes are disassembled upon completion of synthesis before replication is initiated at the segregated copies of *oriC* (Figure 3B). Time-lapse analysis of both *oriC^+^ oriZ^+^* and *oriC^+^ oriX^+^* cells showed that both origins are active, as shown before for *oriC^+^ oriZ^+^* cells [19], ruling out that either *oriX/oriZ* or *oriC* fire independently but with similar frequencies.

### 3.3. Termination and Replication–Transcription Conflicts in Double-Origin Cells

Replication initiated at *oriX* and proceeding counter-clockwise will reach the termination area much earlier than forks coming from *oriC* and, consequently, forks will be blocked at the *terA*/Tus complex—the first *ter*/Tus complex encountered in blocking orientation—which results in the clearly visible step of the replication profile at *terA* (Figure 3(AII)). A similar step is observed in *oriC^+^ oriZ^+^* cells at *terC/B* (Figure 3(AIII)) [18]. Deletion of *tus* in *oriC^+^ oriX^+^* cells enabled replication forks to proceed into the opposite replichore, resulting in a symmetrical replication profile (Figure 3(AIV)). The arithmetic midpoint between *oriC* and *oriX* is at position 1.010 Mbp, close to the measured low point of the LOESS regression at 0.991 Mbp (Table S1). Thus, even if leaving the termination area in a direction opposite to normal, forks appear to proceed with similar speed. In line with this, the introduction of an rpo* point mutation, which decreases the stability of transcribing RNA polymerase (RNAP) complexes [29], did not significantly change the location of the low point of the replication profile (Figure 3(AV)), suggesting that problems associated with replication–transcription encounters must be similar for both replication machineries.

**Figure 4:**
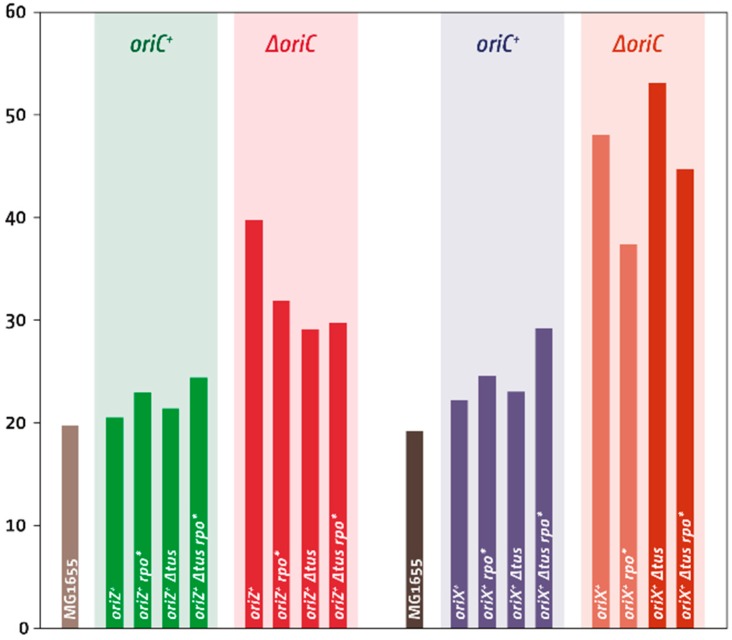
Comparison of doubling times of *oriZ^+^* constructs, as reported in [18], and *oriX^+^* constructs (this study). The presence or absence of *oriC* is highlighted above each group of strains. The ectopic origin and all other genotype details are identified for each strain individually. The two *ΔoriC oriX^+^* constructs, identified by a lighter colour, contained large chromosomal inversions (see main text for details). All doubling times were determined by measuring viable titres of cultures grown in Luria broth (LB) (see Material and Methods for details).

Doubling times of all *oriC^+^ oriX^+^* constructs followed trends that were similar to our previous observations in *oriC^+^ oriZ^+^* cells (Table 2 and Figure 4). Introduction of *oriX* mildly slowed the doubling time, indicating that integration of a second replication origin interferes in some way with the fast growth observed in wild-type cells. An rpo* point mutation was shown before to slow growth [18] and, consequently, a slower doubling time is seen in oriC^+^ oriX^+^ rpo* cells (Table 2 and Figure 4). A *tus* deletion had little effect, but a combination of *Δtus* and rpo* resulted in the slowest doubling time (Table 2 and Figure 4).

### 3.4. Deletion of oriC in Double-Origin Mutants Triggers Chromosomal Rearrangements

**Figure 5:**
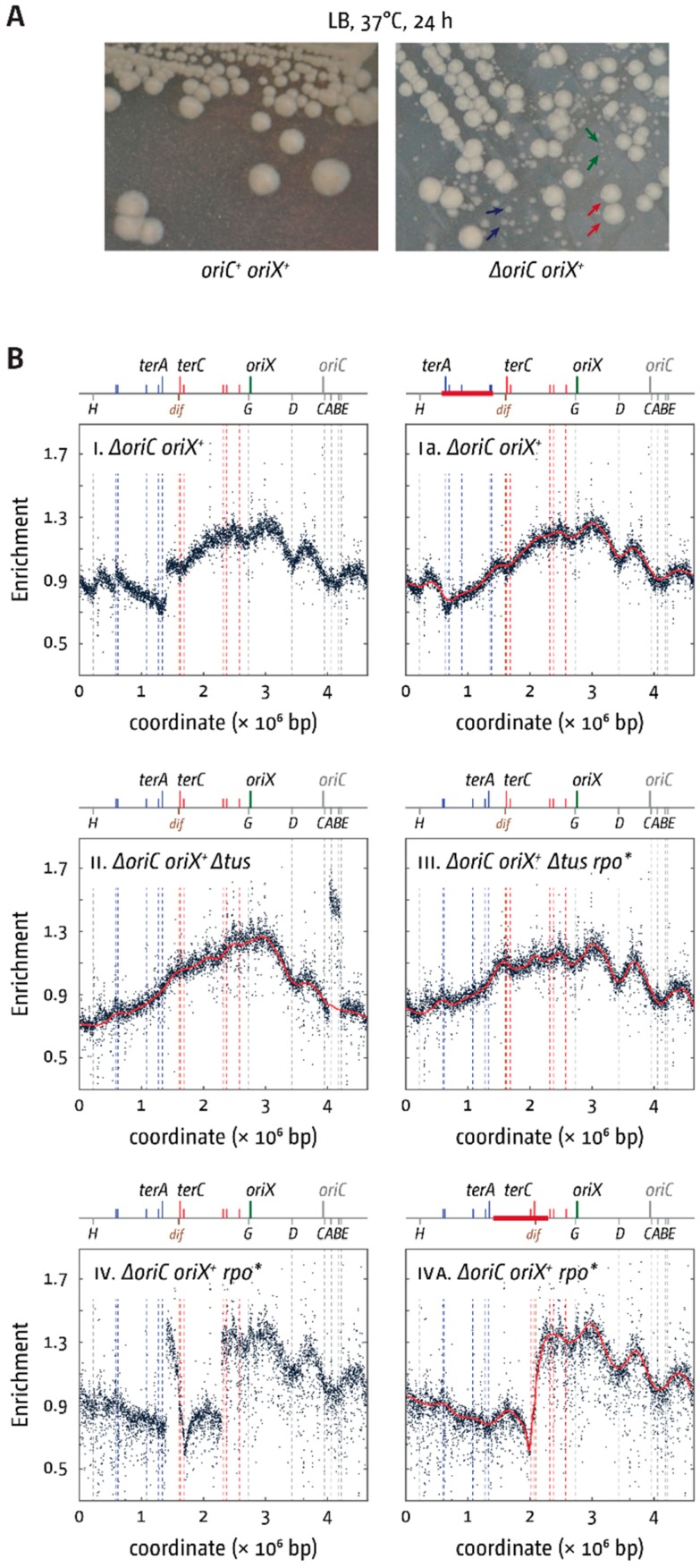
Growth and replication profiles of *E. coli* cells replicating from a single ectopic replication origin. (**A**) Large colony variants due to accumulation of suppressor mutations in *ΔoriC oriX^+^* cells. Shown is a streak to single colonies of an overnight culture of both constructs. While an *oriC^+^ oriX^+^* strain shows largely uniform colony sizes with only some variation due to colony density, a *ΔoriC oriX^+^* construct shows small, medium, and large colonies, as highlighted by green, blue, and red arrows, respectively. The strains used were JD1181 (*oriC^+^ oriX^+^*) and JD1187 (*∆oriC oriX^+^*). (**B**) Replication profiles of *E. coli* cells with a single ectopic replication origin. Shown is the MFA of E. coli ΔoriC oriX^+^, ΔoriC oriX^+^ ∆tus, ΔoriC oriX^+^ rpo*, and ΔoriC oriX^+^ ∆tus rpo* cells. The number of reads (normalised against reads for a stationary phase wild-type control) is plotted against the chromosomal location. A schematic representation of the *E. coli* chromosome showing positions of *oriC* and *oriZ* (green line) and *ter* sites (above), as well as *dif* and *rrn* operons *A–E*, *G*, and *H* (below), is shown above the plotted data. Clear discontinuities of the profiles can be seen in panels i, ii, and iv. For panels i and iv, these are due to large inversions, as highlighted by the continuous replication profile that results if the area highlighted in red in the schematic representation of the chromosome is inverted. The strains used were JD1187 (*ΔoriC oriX^+^*), JD1208 (*ΔoriC oriX^+^ ∆tus*), JD1197 (ΔoriC oriX^+^ rpo*), and JD1209 (ΔoriC oriX^+^ ∆tus rpo*).

In *ΔoriC oriX^+^* cells, only *rrnD* is replicated in an orientation opposite to normal, together with a cluster of ~30 genes encoding for ribosomal proteins. However, *ΔoriC oriX^+^* cells had a growth rate even slower than that of *ΔoriC oriZ^+^* cells (Table 2 and Figure 4) [18] and rapidly accumulated fast-growing suppressor mutations (Figure 5A). Given our experience of suppressor accumulation in *ΔoriC oriZ^+^* cells, we were vigilant for spontaneous suppressor mutations arising whilst generating *ΔoriC oriX^+^* constructs. Nevertheless, our *ΔoriC oriX^+^* construct contained a gross chromosomal rearrangement (GCR), inverting an ~820 kb fragment of the chromosome that spans from IS5 at 575 kb to IS5 at 1394 kb (Figure 5B(I,Ia); Figure S3 for PCR verification of the inversion). This inversion spans all restrictive *ter* sites (*terA*, *D*, *E*, *H*, and *I*) and flips them into permissive orientation, thereby allowing forks to leave the termination area. While the previously reported inversion that realigned replication and transcription in *ΔoriC oriZ^+^* cells acted as a very efficient suppressor of the slow-growth phenotype [18], the *ΔoriC oriX^+^* construct containing the inverted *ter* sites (*ΔoriC oriX^inv^*) grew slowly (Table 2 and Figure 4), suggesting that additional effects must interfere with efficient chromosome duplication. We suspect that *ΔoriC oriX^+^* cells without the GCR have an even longer doubling time or might potentially be inviable.

The doubling time of *ΔoriC oriX^+^ Δtus* cells was roughly comparable to that of our *ΔoriC oriX^+^* construct carrying the GCR, in line with the replication fork trap not being active in both backgrounds (Table 2 and Figure 4). The doubling time of the *ΔoriC oriX^+^ Δtus* construct was markedly longer than that of the corresponding *ΔoriC oriZ^+^ Δtus* construct (Table 2 and Figure 4), and the replication profile of *ΔoriC oriX^+^ Δtus* cells (Figure 5(BII)) revealed a discontinuity that indicates a duplication of a 175 kb stretch spanning the *rrn* operons *A* and *B*. This GCR turned out to be a spontaneous mutation in the culture grown for the preparation of gDNA, but not in our stock culture, as a second replication profile showed no GCR (Figure S4). This suggests that the measured doubling time (Table 2 and Figure 4) was correctly determined.

To determine the impact of replication-transcription conflicts that occur when part of the chromosome is replicated in an orientation opposite to normal, a ΔoriC oriX^+^ rpo* construct was generated. This construct indeed showed a faster doubling time (Table 2 and Figure 4), but it contained yet another GCR. An 895 kb section of the chromosome spanning from IS5 at 1394 kb to IS5 at 2288 kb was inverted (Figure 5B(IV,IVA); see Figure S3 for PCR verification of the inversion). In this case, the GCR was observed in two independent MFAs, suggesting that it has arisen during the construction process. Its presence prevents a detailed analysis. However, the doubling time of the ΔoriC oriX^+^ rpo* construct carrying the inversion is faster than those of *ΔoriC oriX^inv^* and *ΔoriC oriX^+^ Δtus* (Table 2), suggesting that the rpo* mutation still improves growth. Indeed, introduction of an rpo* point mutation into *ΔoriC oriX^+^ Δtus* cells resulted in a decrease of the doubling time (Table 2), in line with the idea that replication-transcription conflicts contribute to the slow-growth phenotype of *ΔoriC oriX Δtus* cells. The ΔoriC oriX^+^ Δtus rpo* construct is the only construct without GCRs, similar to ΔoriC oriZ^+^ Δtus rpo* cells, in which suppressor accumulation is markedly reduced [18]. However, the growth rate of ΔoriC oriX^+^ Δtus rpo* cells is still substantially slower than that of the equivalent ΔoriC oriZ^+^ Δtus rpo* construct (Table 2 and Figure 4), further supporting the idea that a number of factors influence the doubling time in *oriX^+^* cells.

### 3.5. Replication Initiation in Cells with a Triple-Origin Chromosome

**Figure 6:**
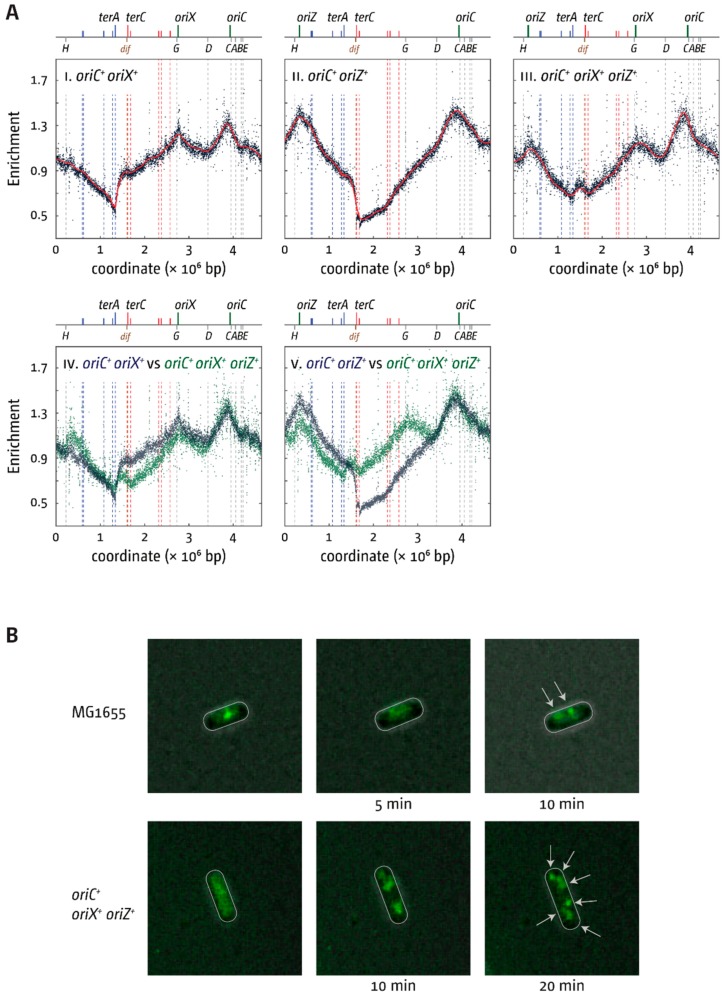
Replication dynamics in *E. coli* cells with one and two ectopic replication origins. (**A**) MFA in *E. coli oriC^+^ oriZ^+^, oriC^+^ oriX^+^*, *and oriC^+^ oriX^+^ oriZ^+^* cells. The number of reads (normalised against reads for a stationary phase wild-type control) is plotted against the chromosomal location. A schematic representation of the *E. coli* chromosome showing positions of *oriC, oriX*, and *oriZ* (green lines) and *ter* sites (all above), as well as *dif* and *rrn* operons *A–E*, *G*, and *H* (all below), is shown above the plotted data. The strains used were JD1181 (*oriC^+^ oriX^+^*), RCe504 (*oriC^+^ oriZ^+^*), and JD1333 (*oriC^+^ oriX^+^ oriZ^+^*). (**B**) Visualisation of replisomes (Ypet-DnaN) in wild-type and *oriC^+^ oriX^+^ oriZ^+^* triple-origin cells. Cells were grown in M9 minimal salts medium with 0.2% glucose and transferred onto a thin agarose pad of the same medium on a microscopy slide (see Material and Methods). Slides were transferred into a chamber heated to 37 °C and fluorescent foci in single cells tracked over time. The strains used were AS1062 (*ypet-dnaN*) and RCe753 (*oriC^+^ oriX^+^ oriZ^+^ ypet-dnaN*).

We wanted to investigate whether an *E. coli* chromosome with three active origins could be constructed. In *oriC^+^ oriX^+^ oriZ^+^* cells, defined areas would be replicated opposite to normal, thereby causing some difficulties, but replication should be less asymmetric than in double-origin cells. Construction of an *oriC^+^ oriX^+^ oriZ^+^* construct was easily achieved. However, the doubling time of this construct was longer than that of both wild-type and double-origin cells (Table 3), and the replication profile revealed a surprising skew in origin usage (Figure 6).

**Figure 7:**
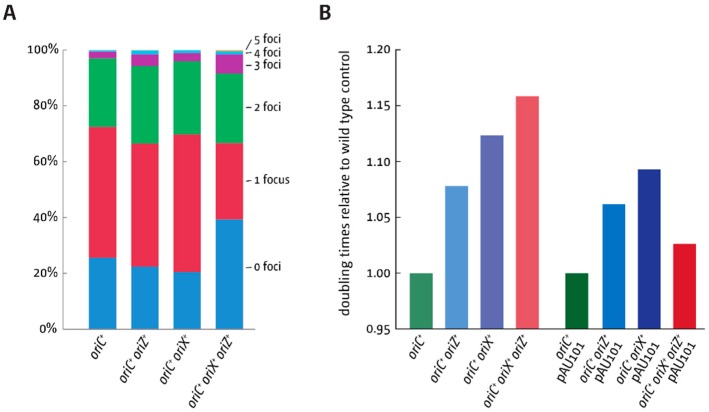
Replisome numbers and doubling times of cells with one and two ectopic replication origins. (**A**) Replisome numbers (YPet-DnaN) in wild-type, *oriC^+^ oriX^+^*, *oriC^+^ oriZ^+^*, and *oriC^+^ oriX^+^ oriZ^+^* cells. A minimum of 300 cells from at least three independent experiments were analysed per strain. Shown are the average focus counts per strain and focus class. The strains used were AS1062 (*ypet-dnaN*), RCe749 (*oriC^+^ oriZ^+^ ypet-dnaN*), RCe751 (*oriC^+^ oriX^+^ ypet-dnaN*), and RCe753 (*oriC^+^ oriX^+^ oriZ^+^ ypet-dnaN*). (**B**) Doubling times of *E. coli* cells with one or two ectopic replication origins in the presence and absence of an additional copy of the *dnaA* gene expressed from a low copy number plasmid from its native promoter. All doubling times were determined by measuring viable titres of cultures grown in LB broth (see Material and Methods for details). Changes in doubling times relative to wild-type cells are shown due to the fact that the presence of ampicillin necessary for plasmid selection causes a mild change in doubling times (see Table S2). The strains used were MG1655, RCe504 (*oriC^+^ oriZ^+^*), JD1181 (*oriC^+^ oriX^+^*), and JD1333 (*oriC^+^ oriX^+^ oriZ^+^*) in the presence or absence of plasmid pAU101 (see Supplementary Methods), as indicated.

*oriC* showed the highest peak height, while the peak heights of both *oriZ* and *oriX* were reduced (Figure 6(III)). As replication profiles only give an indication of origin usage within a population of cells, time-lapse fluorescence microscopy of *oriC^+^ oriX^+^ oriZ^+^* cells carrying YPet-DnaN was used to investigate whether there are cells in which all three origins can be active. While the signal in double-origin cells produced defined foci (Figure 3B), the signal in triple-origin cells was less defined. In addition, the close proximity of multiple and less-defined foci made differentiation with conventional fluorescence microscopy very difficult. Nevertheless, in some cells, three separate foci were observed, suggesting that all three origins can be active at least in a fraction of cells (Figure 6B). Given the resolution limit of conventional fluorescence microscopy and the fact that the β-sliding clamp remains bound to DNA for some time after the replisome has passed [30,31], we did not attempt a detailed analysis of foci dynamics in cells, as this is unlikely to result in meaningful data. However, foci numbers in snap shots of cells in the exponential phase grown in M9 minimal medium with 0.2% glucose were analysed. Fluorescent DnaN foci per cell were then counted (Figure 7A). Overall, only a minor increase in the number of DnaN foci per cell was observed both in *oriC^+^ oriZ^+^* and *oriC^+^ oriX^+^* cells, despite the fact that time-lapse analysis shows clearly that both origins are active (Figure 3B). We believe the main reason for this is the short presence of multiple replisomes. Upon initiation of replication, one replisome coming from *oriC* will replicate a relatively short stretch of 500 kb before it is met by a replisome coming from the ectopic origin. If replication proceeds with the reported 650–1000 nt·s^−1^ [32], forks will fuse after 10–12 min and disassemble, leaving two forks that move in the opposite directions, the same number as in wild-type cells. Thus, in asynchronously growing cultures, only a small fraction of cells will show an increased number of replisomes, which, together with the limited resolution, explains the very moderate shift in foci numbers.

One interesting feature of triple-origin cells is the increase in cells with no foci, while both *oriC^+^ oriX^+^* and *oriC^+^ oriZ*^+^ cells show a decrease in comparison to wild-type cells. One explanation for this effect might be a limitation of initiation of DNA replication in triple-origin cells. It was reported before that multiple chromosomal locations, including the *datA* locus, bind the DnaA initiator protein with high affinity [33]. Upon initiation of chromosome replication, the duplication of these regions will act as a sink for DnaA, thereby reducing the concentration of free DnaA protein in the cell [34], which limits initiation of replication [35,36].

Levels of DnaA are clearly high enough to allow simultaneous initiation at two independent copies of the origin (Figure 3A,B) [18,19]. However, a third copy might cause the concentration of free DnaA to drop below the threshold level for initiation for longer, thereby limiting initiation of replication and thus leading to an increased fraction of cells with zero foci. This effect might also explain why triple-origin cells grow more slowly than both double-origin constructs (Table 3 and Figure 7B). To test whether this was the case, a low copy number plasmid carrying a copy of *dnaA* under its native promoter was introduced into these cells and the doubling times measured. An increased *dnaA* copy number caused only minor reductions of the doubling time of double-origin cells, but triple-origin cells show a marked reduction, in line with the idea that the concentration of free DnaA becomes limiting (Figure 7B and Table S2).

Finally, we wanted to investigate growth of a *ΔoriC oriX^+^ oriZ^+^* construct. A *ΔoriC oriX^+^ oriZ^+^* construct has a symmetrical replichore arrangement, but forks coming both from *oriX* and *oriZ* will still replicate one-quarter of the chromosome in an orientation opposite to normal, which would be expected to impose problems. In line with this assumption, the deletion of *oriC* increased the doubling time to 35.3 min (Table 3). However, the doubling time of *ΔoriC oriX^+^ oriZ^+^* cells is still significantly quicker than that of *ΔoriC oriX^+^* cells, suggesting that the presence of *oriZ* alleviates some of the problems that occur in *ΔoriC oriX^+^* cells.

## 4. Discussion

Previously, we investigated replication dynamics in cells in which an ectopic origin termed *oriZ* was integrated in the right-hand replichore [18,19]. In this study, we attempted to integrate ectopic replication origins at different locations in the left-hand replichore. We hypothesised that replication-transcription conflicts should be less severe, as the left-hand replichore contains less highly transcribed *rrn* operons (Figure 1). We were surprised to find that the attempted integration of *oriY* at 3.55 Mbp into the chromosome—a position where no highly transcribed *rrn* operons are encountered head-on—only resulted in constructs in which the *oriC* core sequences were truncated (Figure 2), despite the use of PCR products with the correct length. The truncations differed in all constructs analysed, suggesting that they are spontaneous mutations. If so, this indicates strongly that integration of an active origin in this precise location is toxic, while the general integration of sequences such as the antibiotic resistance marker is not. This result rules out that inactivation of *malT* itself is harmful to cells for some reason or that the integration of this fragment somehow activates a cryptic gene that might be toxic for cells. Indeed, it was reported before that integration of an ectopic replication origin resulted in silencing of the native *oriC* [17], supporting the idea that the activity of two origins in close proximity might cause problems for cells.

In contrast, integration of *oriX* into *pheA* was unproblematic, and replication profiles, as well as fluorescence microscopy analysis, confirmed that, in *oriC^+^ oriX^+^* cells, both origins are active and fire with similar frequencies (Figure 3; Figure S2), as observed for *oriC^+^ oriZ^+^* cells [18,19] (Figure 3).

### 4.1. Termination and Replication-Transcription Conflicts in Double-Origin Strains

The features of the replication profile of *oriC^+^ oriX^+^* cells were similar to the replication profiles of *oriC^+^ oriZ^+^* cells [8,18]. The innermost *ter* sites—*terA* and *terD*—stop synthesis coming from *oriX* efficiently, causing a marked asymmetry in the termination area (Figure 3). The impact of *ter*/Tus complexes is highlighted in particular by the ~800 kb inversion found when we attempted to generate a *ΔoriC oriX^+^* construct. This inversion flipped all *ter* sites of the left-hand replichore into the permissive orientation for replication coming from *oriX,* thereby effectively inactivating the replication fork trap in this replichore (Figure 5). Thus, the situation in *ΔoriC oriX^+inv^* cells should be similar to the situation in *ΔoriC oriX^+^ Δtus* cells, and, indeed, *ΔoriC oriX^+inv^* and *ΔoriC oriX^+^ Δtus* cells had similar doubling times (Table 2 and Figure 4). Since no “clean” *ΔoriC oriX^+^* construct was generated, we currently do not know whether the inactivation of *tus* acts as a suppressor of the slow-growth phenotype of *ΔoriC oriX^+^* cells. However, it is likely that the doubling time of *ΔoriC oriX^+^* cells is even longer. In this case, both the deletion of *tus* and the inversion of all blocking *ter* sites act indeed as suppressor mutations of the slow-growth phenotype of *ΔoriC oriX^+^* cells, as observed in *ΔoriC oriZ^+^* cells (Table 2 and Figure 4) [18].

Our results show that the growth rate of *ΔoriC oriX^+^ Δtus* cells is considerably slower than that of the equivalent *ΔoriC oriZ^+^ Δtus* construct (Table 2 and Figure 4), suggesting that replication in *ΔoriC oriX^+^ Δtus* cells has to deal with other serious problems that do not apply in the same way to *ΔoriC oriZ^+^ Δtus* cells. One contributing factor might be head-on replication-transcription encounters, and the doubling time of ΔoriC oriX^+^ Δtus rpo* cells is indeed reduced in comparison to *ΔoriC oriX^+^ Δtus* cells (Table 2 and Figure 4). Given that an rpo* point mutation itself slows the doubling time of wild-type cells [18], the real effect is likely to be even stronger than the difference immediately obvious from the direct comparison of *ΔoriC oriX^+^ Δtus* cells with and without rpo*. However, the fact that the doubling time of ΔoriC oriX^+^ Δtus rpo* cells is significantly longer than that of ΔoriC oriZ^+^ Δtus rpo* cells (Table 2 and Figure 4) further supports the idea that additional factors must interfere with successful and efficient chromosome duplication in *ΔoriC oriX^+^* cells.

### 4.2. Large Chromosomal Rearrangements in Double-Origin Cells

A clue as to which additional factors might interfere with DNA replication in *ΔoriC oriX^+^* cells might come from a spontaneous rearrangement observed in one of our *ΔoriC oriX^+^ Δtus* cultures, duplicating the chromosomal stretch containing *rrn* operons *A* and *B* (Figure 5(BII)). The location of important genetic elements relative to the origins and the resulting gene dosage effect was described before [37]. The *rrn* operons *CABE* and *D* are all located in close proximity to the replication origin, ensuring an increased copy number in fast-growing cells (Figure 3A) [37]. In contrast, shifting the origin from its original to the *oriX* location results in a much-reduced copy number, especially of the *rrn* operons *CABE* and *H* (Figure 5(BI)). This effect is specific to *oriX* due to its distance to all *rrn* operons, with the exception of *rrnG*. The location of *oriZ* is in close proximity to *rrn* operon *H* and the *rrnCABE* cluster (Figure 1), providing a potential explanation for why *ΔoriC oriZ^+^* cells struggle less. In addition, the inversion found in *ΔoriC oriZ^+^* cells not only realigns replication and transcription, but also brings the *rrnCABE* cluster in close proximity of *oriZ* [18], explaining perhaps why this particular inversion is such an efficient suppressor of the slow-growth phenotype despite a persisting replication asymmetry. It is tempting to speculate that *ΔoriC oriZ^+^ Δtus* cells containing the duplication of *rrnA* and *rrnB* will be able to grow faster. However, as this duplication was spontaneously acquired in a culture for gDNA extraction and was only revealed after sequencing, it was not possible to measure whether it conferred a growth advantage. Indeed, other effects might contribute. It was shown before that deletion of *rrn* operons affects the growth rate of cells only moderately [38,39]. However, in a recent study, a duplication of a similar location was observed as a suppressor of the severe growth defect of cells lacking the DnaA regulatory inactivator Hda [40]. The suppression of the slow-growth phenotype of *Δhda* cells was found to be the increased gene dosage for DNA polymerase I (*polA*) [40]. This or other similar effects might be important contributors in *oriX* cells.

The large number of GCRs observed as part of our studies fits well with previous reports of a surprising number of rearrangements in a limited set of *E. coli* samples, including a duplication of the *rrn* operons *A*, *B*, and *E* [41], highlighting a surprising degree of plasticity of the *E. coli* chromosome. Rearrangements and especially duplications are among the most frequent mutational events [41,42]. However, unless they confer an immediate advantage, they will be rapidly lost because of a fitness cost [41]. Given the slow growth of *ΔoriC oriZ^+^* cells and the robust suppression by the inversion, the isolation of the GCR observed is not much of a surprise, as it will outgrow the original construct very rapidly. We assume that a similar argument can be made for the GCR observed in our *ΔoriC oriX^+^* construct (Figure 5). Perhaps the biggest surprise is the inversion observed in ΔoriC oriX^+^ rpo* cells. An 895 kb section of the chromosome spanning from IS5 at 1394 kb to IS5 at 2288 kb was inverted (Figure 5B(IV,IVA); see Figure S3 for PCR verification of the inversion). This inversion not only brings the *ter* sites *C* and *B* in close proximity of *oriX*, but also switches them to the restrictive orientation, forcing the replication fork coming from *oriX* travelling in the normal orientation to stop after 650 kb. The remaining 4000 kb of the chromosome have to be replicated by the clockwise replication fork. If this inversion acts as a suppressor mutation, then it must alleviate a yet unidentified replication stress, but the replication profile gives little clue as to what this stress might be. However, the doubling time of the ΔoriC oriX^+^ rpo* construct carrying the inversion is quicker than the doubling time of *ΔoriC oriX^inv^* and *ΔoriC oriX tus* (Table 2), suggesting that the rpo* mutation does indeed improve growth, despite the effect of the highly asymmetric replichore arrangement.

It is noteworthy that two of the inversions found in this study have specifically arisen at IS5 elements, which provide large stretches (~1.2 kb) of homology. These insertion elements (IS elements) allow for relatively frequent large chromosomal rearrangements to occur that clearly can efficiently alleviate problems during replication and other cellular processes. Indeed, it was shown that the systematic deletion of all IS elements caused a robust genetic stabilisation, with a 75% decrease of the mutation rate determined in this particular study [43], demonstrating their contribution towards the observed plasticity of the genome.

### 4.3. Replication in Cells with Three Functional Replication Origins

The replication profiles of our triple origin construct provide further evidence of how finely balanced the replication parameters of the *E. coli* chromosome are. While our fluorescence microscopy studies show that all three origins can be active in some of the cells (Figure 6B), the replication profile revealed that the peak height of both ectopic origins was significantly reduced (Figure 6). This suggests that all three origins being simultaneously active is probably a rare event. It is likely that in a fraction of cells, only two of the three origins might be active, one of which almost always is the native *oriC*. In *oriC^+^ oriZ^+^* and *oriC^+^ oriX^+^* cells, both the ectopic and the native origin fire with similar frequency (Figure 3) [18,19], suggesting that both are equivalent. Apparently, this changes in a triple-origin background, even though the reason for this effect is not known. The reduction of the doubling time of triple-origin cells in which an additional copy of *dnaA* was introduced via a low copy number plasmid (Figure 7B) suggests that three copies of the origin per cell generate an environment where, at least in some cells, the threshold level of DnaA necessary for efficient origin initiation is not reached for some time. This causes a delay of initiation of all origins in a fraction of cells, which explains the increased level of cells in which no replisomes are observed (Figure 7A). Thus, our data are in line with the idea that a delay of origin firing contributes to the slow doubling time of triple-origin cells.

Nevertheless, if all origins were equivalent, there should be an equal reduction of peak heights of all three origins, which was not observed. The *oriC* peak is significantly higher, demonstrating that the *oriC* sequence in its native location has the highest capacity for being active. Indeed, bacterial chromosomes with a single origin are the norm [3], despite the fact that the resulting long replichores require replication machineries with very high speed and accuracy in comparison to DNA synthesis in eukaryotic cells. It was suggested that the genes flanking the origin sequence might influence origin activity [44], explaining why cells carrying a 5 kb *oriC* region stretch, as developed in the Sherratt lab [19], are active, whereas smaller fragments are not [44]. It is possible that an even larger fragment of the chromosome is required for full functionality, which might explain the reduced activity of both *oriX* and *oriZ* in our *oriC^+^ oriX^+^ oriZ^+^* construct (Figure 6). However, the toxicity of the 5 kb origin fragment integrated into the *malT* gene strongly argues that this assumption is too simple, as there appear to be strong effects relating to the position of multiple origins relative to each other, the precise location of an origin within the cell, or the combination of multiple effects.

**Figure 8:**
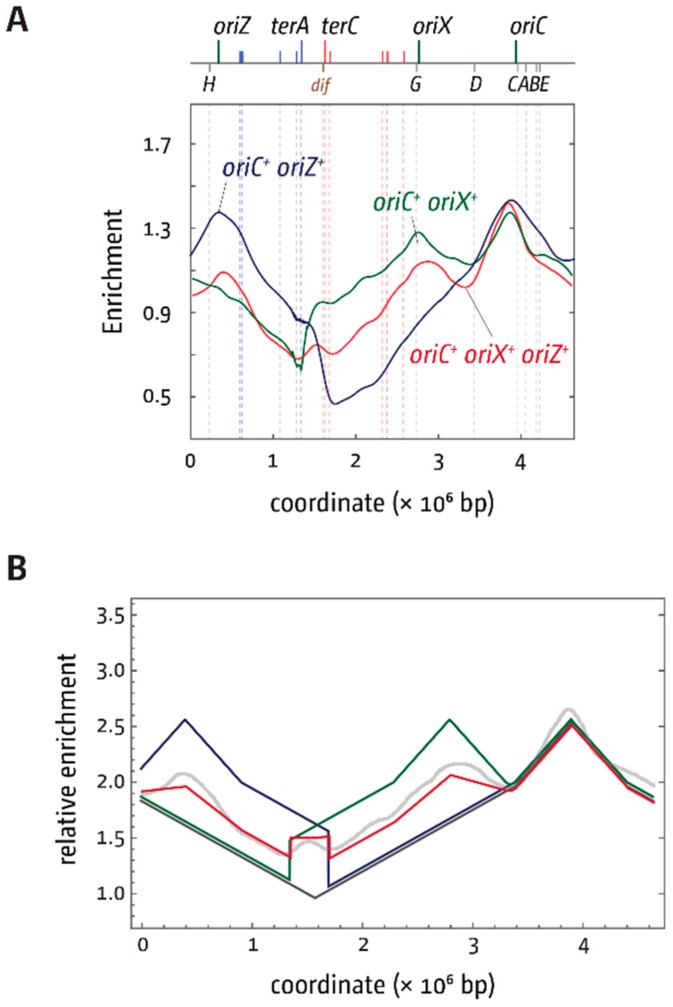
Comparative analysis of replication profiles of *E. coli* cells with two and three replication origins. (**A**) Shown is a combination of the LOESS regression profiles for *oriC^+^ oriZ^+^* (blue), *oriC^+^ oriX^+^* (green), and *oriC^+^ oriX^+^ oriZ^+^* (red) cells, as shown in Figure 6. (**B**) Mathematical model fitting for *oriC^+^ oriZ^+^* (blue), *oriC^+^ oriX^+^* (green), and *oriC^+^ oriX^+^ oriZ^+^* (red) cells. The modelling for *oriC^+^ oriX^+^ oriZ^+^* (red) assumes that *oriC* and *oriX* are active in 50% of cells, and *oriC* and *oriZ* are active in 40% of cells, based on the peak heights in the replication profiles. The LOESS regression curve for *oriC^+^ oriX^+^ oriZ^+^* cells is shown in light grey, as shown in Figure 6. See text for further details.

We were intrigued to find what looks like a peak of over-replication within the termination area. Similar peaks were reported in cells lacking RecG helicase [23], RNase HI [45,46], and other proteins [8,47,48]. We have postulated that the fusion of two replisomes in the termination area results in intermediates which require processing by proteins such as RecG helicase and 3′ exonucleases [8,23,49,50,51,52], the absence of which results in substantial amounts of over-replication in the termination area. However, all the above proteins are fully functional in our triple-origin construct, making it unlikely that the peak is a similar type of over-replication. In fact, the peak can be fully explained if replication is initiated at two of the three origins in a significant fraction of cells. In *oriC^+^ oriZ^+^* cells, marker frequency is high throughout the termination area, with a marked decrease at *terC/B* (Figure 3(AIII)). In *oriC^+^ oriX^+^*, the opposite is the case. Marker frequency is again high throughout the termination area, with a marked decrease at *terA/D* (Figure 3(AII)). If in triple-origin cells a significant fraction of cells only uses two origins, as the replication profile of triple-origin cells suggests, then the replication profile of triple-origin cells should be formed by the superposition of the two profiles of *oriC^+^ oriX^+^* and *oriC^+^ oriZ^+^* cells (Figure 8A).

In both, the marker frequency is high in the middle of the termination area, while the areas around *terC/B* and *terA/D* should be reduced because of the marked decrease in one fraction of cells (Figure 8A). We exploited mathematical modelling of whole genome replication [53] (see Supplementary Methods) to predict the replication profile within a population of cells where either *oriC* and *oriX* or *oriC* and *oriZ* are active. In our modelling, we assumed a constant fork speed once forks are established. The periodicity of origin firing was estimated from our experimental data. For simplicity, *ter*/Tus complexes were treated as a hard stop to replication. While the resulting modelled replication profile lacks the complexity of our data sets (Figure 8B), it fits overall well with the population-based replication profile and shows a clear peak in the termination area, as predicted. This supports the idea that this peak is indeed caused by the presence of defined fractions within the overall population, rather than actual over-replication of the termination area.

As the replication profiles of cells lacking RecG helicase or 3′ exonucleases have been generated from a similar population-based approach [23,45,48], it could be suggested that the peaks observed might be resulting from a similar superposition of different populations. Indeed, it was recently shown that the sharp loss of sequences corresponding to the terminus area in the replication profile of a *recB* mutant strain stems only from a defined fraction of cells [54]. However, the presence of synthesis in the termination area was confirmed using different experimental approaches [52], and we were able to demonstrate that cells lacking RecG helicase can tolerate the inactivation of *oriC* as long as the termination area is inactivated by deletion of *tus* and replication–transcription encounters are alleviated by the presence of an rpo* point mutation [23,45]. Thus, there is no doubt that extra synthesis is indeed initiated in the termination area of cells lacking RecG. However, use of the rapidly emerging single-cell approaches [55] will enable an even more refined approach to these aspects of replication and chromosome dynamics.

## 5. Accession Numbers

All relevant raw sequencing data can be accessed at the European Nucleotide Archive (http://www.ebi.ac.uk/ena/data/view/PRJEB19883).

## Figures and Tables

**Table 1:** *Escherichia coli* K-12 strains.

Strain Number	Relevant Genotype ^a^	Source
**General P1 donors**		
WX297	AB1157 *oriZ-<kan>*	[19]
RRL190	AB1157 *<kan>-ypet-dnaN*	[19]
RUC1593	DY330 *pheA::oriX-cat*	This study
**MG1655 derivatives**		
MG1655	F^–^ *rph-1*	[22]
AS1062	*<kan>-ypet-dnaN*	MG1655 × P1.RRL190 to Km^r^
JD1181	*ΔlacIZYA pheA::oriX-cat*	TB28 × P1.RUC1593 to Cm^r^
JD1187	*ΔlacIZYA pheA::oriX-cat ΔoriC::kan*	JD1181 × P1.RCe576 to Km^r^
JD1190	rpoB*35 ΔlacIZYA pheA::oriX-cat	N5925 × P1.RUC1593 to Cm^r^
JD1197	rpoB*35 ΔlacIZYA pheA::oriX-cat ΔoriC::kan	JD1190 × P1.RCe576 to Km^r^
JD1203	*ΔlacIZYA pheA::oriX-cat tus1::dhfr*	JD1181 × P1.N6798 to Tm^r^
JD1205	rpoB*35 ΔlacIZYA pheA::oriX-cat tus1::dhfr	JD1190 × P1.N6798 to Tm^r^
JD1208	*ΔlacIZYA pheA::oriX-cat tus1::dhfr ΔoriC::kan*	JD1203 × P1.RCe576 to Km^r^
JD1209	rpoB*35 ΔlacIZYA pheA::oriX-cat tus1::dhfr ΔoriC::kan	JD1205 × P1.RCe576 to Km^r^
JD1332	*ΔlacIZYA pheA::oriX-cat oriZ-<kan>*	JD1181 × P1.WX297 to Km^r^
JD1333	*ΔlacIZYA pheA::oriX-cat oriZ-<kan>*	JD1181 × P1.WX297 to Km^r^
JD1336	*ΔlacIZYA oriZ-<kan>*	TB28 × P1.WX297 to Km^r^
JD1339	*ΔlacIZYA oriZ-<>*	JD1336 × pCP20 to Km^s^ Ap^s^
JD1341	*ΔlacIZYA oriZ-<> pheA::oriX-cat*	JD1339 × P1.RUC1593 to Cm^r^
JD1343	*ΔlacIZYA oriZ-<> pheA::oriX-cat ΔoriC::kan*	JD1341 × P1.RCe576 to Km^r^
JJ1359	*ΔlacIZYA dam1::kan ΔrecG::apra tus1::dhfr*	[23]
N4560	*ΔrecG265::cat*	[24]
N5925	rpoB*35 ΔlacIZYA	[25]
N6798	*ΔrecG265::cat tus1::dhfr*	N4560 × P1.JJ1359 to Tm^r^
RCe504	*oriZ-<cat>*	[18]
RCe576	rpoB*35 oriZ-cat-frt tus1::dhfr ΔoriC::kan ^b^	[18]
RCe749	*oriZ-<cat> <kan>-ypet-dnaN*	RCe504 × P1.AS1062 to Km^r^
RCe751	*ΔlacIZYA pheA::oriX-cat <kan>-ypet-dnaN*	JD1181 × P1.AS1062 to Km^r^
RCe753	*ΔlacIZYA oriZ-<> pheA::oriX-cat <kan>-ypet-dnaN*	JD1341 × P1.AS1062 to Km^r^
TB28	*ΔlacIZYA*	[26]

a—Only the relevant additional genotype of the derivatives is shown. The abbreviations *kan*, *cat*, and *dhfr* refer to insertions conferring resistance to kanamycin (Km^r^), chloramphenicol (Cm^r^), and trimethoprim (Tm^r^), respectively. *frt* stands for the 34 bp recognition site of the FLP/*frt* site-directed recombination system. b—*ΔoriC* refers to a replacement of the entire origin region (754 bp), including DnaA boxes and 13mers, as well as the entire *mioC* gene, by a kanamycin resistance cassette [23].

**Table 2:** Doubling times of *E. coli* strains with an ectopic replication origin in the left replichore.

Strain Background	Doubling Time (min)	SD	r²	Doubling Time *oriZ* Constructs ^a^
MG1655	19.3	±1.7	0.983	19.9
*oriC^+^ oriX^+^*	22.3	±1.2	0.981	20.6
*ΔoriC oriX^+^*	48.1	±5.6	0.969	39.8
*oriC^+^ oriX^+^ Δtus*	23.1	±0.7	0.985	21.5
oriC^+^ oriX^+^ rpoB*35	24.7	±1.5	0.986	23.1
oriC^+^ oriX^+^ Δtus rpoB*35	29.3	±1.9	0.993	24.5
*ΔoriC oriX^+^ Δtus*	53.2	±9.1	0.977	29.2
ΔoriC oriX^+^ rpoB*35	37.5	±8.4	0.980	32.0
ΔoriC oriX^+^ Δtus rpoB*35	44.8	±9.2	0.99	29.8

a—doubling times as reported in [18]. SD: standard deviation

**Table 3:** Doubling times of *E. coli* strains with two ectopic replication origins.

Strain Background	Doubling Time (min)	SD	r²
MG1655	19.6	±1.0	0.999
*oriC^+^ oriZ^+^*	21.0	±0.8	0.997
*oriC^+^ oriX^+^*	21.8	±0.8	0.996
*oriC^+^ oriX^+^ oriZ^+^*	22.7	±2.5	0.994
*ΔoriC oriX^+^ oriZ^+^*	35.3	±2.6	0.990

## Supplementary Materials

The following are available online at http://www.mdpi.com/2073-4425/9/8/376/s1, Table S1: Replication profile minima established by LOESS regression of the replication profiles of *E. coli* strains with one and two replication origins, Table S2: Effect of increased *dnaA* gene dosage on the doubling times in cells with one and two ectopic replication origins, Figure S1: Marker frequency analysis and sample quality of *E. coli* *Δ**oriC oriX+* *Δ**tus rpo** cells following short (30 min) and extended (120 min) deproteinisation via proteolytic digest using proteinase K. The numbers of reads (normalised against reads for a stationary phase wild-type control) are plotted against the chromosomal location. A schematic representation of the *E. coli* chromosome showing positions of *oriC* and *oriX* and *ter* sites (above), as well as *dif* and *rrn* operons *A–E*, *G*, and *H* (below), is shown above the plotted data. The strain used was JD1209 (*Δ**oriC oriX+* *Δ**tus rpo**), Figure S2: Marker frequency analysis of *E. coli oriC+ oriX+* cells following phenol–chloroform extraction of genomic DNA. The numbers of reads (normalised against reads for a stationary phase wild-type control) are plotted against the chromosomal location. A schematic representation of the *E. coli* chromosome showing positions of *oriC* and *oriX* (green line) and *ter* sites (above), as well as *dif* and *rrn* operons *A–E*, *G*, and *H* (below) is shown above the plotted data, Figure S3: PCR verification of chromosomal inversions. (A) Schematic representation of primer binding sites, inversion locations, and the relocation of primer binding sites following specific inversion events. The schematic showing the inversion between IS5 elements at location 575 kb and 1394 kb is shaded in red, the schematic showing the inversion between IS5 elements at 1394 kb and 2288 kb is shaded blue. The wild-type situation is shaded in yellow. Primers have a single letter identifier, which is shown in bold if the binding site is relocated due to an inversion event to highlight their changed position. Location of primer binding sites are not to scale. All expected PCR products are between 3 and 6.5 kb in length. (B) Agarose gel electrophoresis of PCRs with primer combinations probing for the wild-type sequence and chromosomal DNA templates for a wild-type control (yellow), the *Δ**oriC oriX* background carrying the inversion at IS5 elements at 575 kb and 1394 kb (red), as well as the *Δ**oriC oriX rpo** background that carries an inversion at IS5 elements at 1394 kb and 2288 kb. Primer combinations as shown in A are given above each lane. The size of the PCR product for a specific primer combination is indicated by a grey arrow. The + or – indicates whether a PCR product is expected with the template used. Primer combination a and b did not give a PCR product in any PCR attempted. However, PCR products for both primers a and b are obtained if paired with different secondary primers, suggesting that it is the specific combination of a and b that fails to produce a PCR product. An inverted gel image is shown for clarity. (C) Agarose gel electrophoresis of PCRs with primer combinations probing for both inversions and chromosomal DNA templates for a wild-type control (yellow), the *Δ**oriC oriX* background carrying the inversion at IS5 elements at 575 kb and 1394 kb (red), as well as the *Δ**oriC oriX rpo** background that carries an inversion at IS5 elements at 1394 kb and 2288 kb. Primer combinations as shown in A are given above each lane, with a + or – indicating whether a PCR product is expected. An inverted gel image is shown for clarity. All primers that span flanks following both inversion events show a PCR product, confirming both inversion events identified in our replication profiles, Figure S4: Replication profiles of *E. coli* cells with synthesis starting at ectopic replication origins only. (A–B) Marker frequency analysis of *E. coli* *Δ**oriC oriX+* derivatives. The numbers of reads are normalised against reads for a non-growing stationary phase wild-type control and then plotted against the chromosomal location. In this particular run, the noise observed comes from an increased overall level of noise of the entire sequencing run. This is made worse by the fact that the stationary wild-type control was particularly affected by the noise, which introduces this noise into all other samples due to the normalisation. A schematic representation of the *E. coli* chromosome showing positions of *oriC* and *oriX* (green line) and *ter* sites (above), as well as *dif* and *rrn* operons *A–E*, *G*, and *H*, is shown above the plotted data. Inverted regions are highlighted by a red box. Replication profiles in A are obtained from independent experiments, with independently generated chromosomal DNA, library generation, and sequencing runs. Replication profiles in B are reproduced from Figure 5 for comparison. The direct comparison of the *Δ**oriC oriX+* *Δ**tus* replication profile from the first and second run shows a duplication of the *rrnA–B* region present only in the second run, even though cultures for the preparation of genomic DNA were prepared from the same frozen stock (highlighted in red in B and in grey in A), Figure S5. Mathematical modelling of chromosomal replication in *E. coli* with one or multiple origins. (A) Spatiotemporal representation of a replication program for two origins positioned at *x* = 0 and *x* = 0.5. The tops of each inverted red triangle indicate the initiation of replication. Number of genome copies are 1 (white), 2 (yellow), or 4 (red). The difference between two initiation events establishes the periodicity *s*. (B) Age distribution. (C) Mean number of copies. (D) Inferring population composition: overall profile (blue) is a result of 25% of genomes with only origin at *x* = 0 active (red) and 75% of genomes having both origins active. (E) Spatiotemporal representation of the replication program for two asynchronously initiating origins. (F) Mean number of copies for synchronous initiation with 25% of cells firing one origin and 75% firing two origins (blue), and asynchronous initiation with 100% of cells firing two origins but at different times (magenta). (G) Overlay of model predictions for synchronous (blue) versus asynchronous (magenta) initiations and LOESS data of the replication profile of an *oriC+ oriX* strain. Asynchronous initiation predicts a shift of the termination point to the left, while a shift to the right is observed in our experimental data.
