# Supplementary material for: Origins Left, Right, and Centre: Increasing the Number of Initiation Sites in the Escherichia coli Chromosome
Source: Genes (Basel). 2018 Jul 27;9(8):376. doi: 10.3390/genes9080376 (PMC6116050; doi:10.3390/genes9080376)
Supplement: Supplementary file 1 [file genes-09-00376-s001.pdf]

Supplementary Information

Origins Left, Right, and Centre: Increasing the Number of Initiation Sites in the *Escherichia coli* Chromosome

Juachi U. Dimude[1], Monja Stein[1], Ewa E. Andrzejewska[1], Mohammad S. Khalifa[1], Alexandra A. Gajdosova[1], Renata Retkute[2], Ole Skovgaard[3] and Christian J. Rudolph[1,*]

*Corresponding author: christian.rudolph@brunel.ac.uk

[1]Division of Biosciences, College of Health and Life Sciences, Brunel University London, Uxbridge, UB8 3PH, UK

[2]School of Life Science, The University of Warwick, Gibbet Hill Campus, Coventry, CV4 7AL, UK

[3]Department of Science, Systems and Models, Roskilde University, DK-4000 Roskilde, Denmark

SUPPLEMENTARY MATERIAL AND METHODS

Plasmids

Plasmid pAU101 is a derivative of pRC7 [1] carrying the coding sequence for *dnaA*$^+$ including its native promoter. The *dnaA* region was PCR amplified from MG1655 using 5' and 3' primers incorporating *Apa*I sites. The PCR product was cloned into the *Apa*I site within *lacI*^{q} to give pAU101. The coding sequence inserted is transcribed in the same orientation as the disrupted *lacI*^{q} gene. pAU101 fully complements the temperature sensitivity of the *dnaA46* temperature sensitive strains.

Marker Frequency Analysis by Deep Sequencing

Samples from cultures of a strain grown overnight in Luria broth (LB) (see main Material & Methods section) were diluted 100-fold in fresh broth and incubated with vigorous aeration until an A_{600} reached 0.48 at 37 °C. The only exceptions were all *ΔoriC oriX* backgrounds, for which growth was initiated from a single colony from a streak plate to avoid suppressors formed in the overnight culture outgrowing the slow-growing *ΔoriC oriX* derivatives. All cultures were then diluted a second time 100-fold in prewarmed fresh broth and grown again until an A_{600} of 0.48 was reached. Samples from these exponential phase cultures were flash-frozen in liquid nitrogen at this point for subsequent DNA extraction. Growth curves were recorded using the same procedure (see below), demonstrating that cultures grown to an A_{600} of 0.48 did not show any sign of transition into stationary phase. For wild-type cells, incubation of the remaining culture was continued until several hours after the culture had saturated and showed no further increase in the A_{600}. A further sample (stationary phase) was frozen at this point. For all samples shown in the main Figures of this work, DNA was then extracted using the GenElute Bacterial Genomic DNA Kit (Sigma-Aldrich, St. Louis, MO, USA), using a 30 min proteinase K digest at 55°C, as indicated in the manufacturer protocol (see below for limitations of this procedure). Marker frequency analysis was performed using Illumina HiSeq 2500 sequencing (fast run) to measure sequence copy number. FastQC was used for a basic metric of quality control in the raw data. Bowtie2 was used to align the sequence reads to the reference. Samtools was used to calculate the enrichment of uniquely mapped sequence tags in 1 kb windows for an exponentially growing (replicating) sample relative to a non-replicating stationary phase wild-type sample to correct for differences in read depth across the genome and to allow presentation of the data as a marker frequency, as described previously [2–4].

For presentation of the data as a marker frequency replication profile, the raw read counts for each construct were divided by the average of all read counts across the entire genome to correct for the somewhat different absolute numbers of aligned reads in the various samples. The normalised read count values for each exponentially growing sample were then divided by the corresponding normalised read count value from a stationary (non-

replicating) sample. This division cleans the raw data significantly, because data points which are outliers caused by technical aspects (precise sequence environment interfering with library preparation or similar issues) will be similarly distorted both in the exponential and the stationary samples. However, while true in principle, we have observed that there can be variations specifically in these noisy data points even within a single batch of samples processed in parallel. If the absolute sequence reads of the genome fragments causing the noisy data points in a sample are underrepresented in comparison to the same fragments in the stationary phase sample, then the division process described above causes all of these data points to skew below the position of the neighbouring data points. In contrast, if the absolute sequence reads of the fragments are higher than the sequence reads in the stationary control, then the same division process causes all of these data points to skew above the position of the neighbouring data points. An example of this effect can be seen in Figures 3A and 3B. While the sample in panel iii shows no skew, indicating that noise both in the exponential sample and the stationary sample are of a similar level, the sample in panel v shows a clear skew of all noisy data points below the level of neighbouring data points, while the sample in panel vi shows a skew above the level of neighbouring data points. We do not currently know what is causing these variations, even though we have run extensive tests to try to identify their cause. From our tests, we suspect that a combination of factors, including quality of genomic DNA preparation and library generation, contributes to this effect. Whatever the reason, these problems affect mostly the noise and do not obscure the general trend of the bulk of the data points.

We have by now identified another effect that is specifically caused by the quality of the genomic DNA. The genomic DNA extraction via the GenElute Bacterial Genomic DNA Kit (Sigma-Aldrich) requires a 30 min proteolytic digest with proteinase K. This digestion step is not sufficient to fully remove all proteins in the sample. As a consequence, some partially digested or undigested proteins remain bound to DNA fragments. As part of the following column purification procedure, these proteins, including the bound DNA, are removed. This causes areas of the chromosome in which proteins are tightly bound (*ter*/Tus complexes are one example) or which are very frequently bound by proteins (highly transcribed areas such as the *rrn* operons) to be under-represented in the genomic DNA preparation, leading to small dips in the profile. Examples can be seen in Figure 5. In Figure 5B panel i/ia, clearly dips of the profile can be seen at all *rrn* operons and at some of the *ter* sites. As shown in Figure S1, these dips are much reduced if the proteolytic digest of the samples is extended to 2 h.

Mathematical Modelling

We used the DNA replication modelling described in Retkute et al. [5]. Our modelling has the following assumptions: (i) the length of the chromosome is normalised by half of its length with *oriC* positioned at $x = 0$ (i.e., the length unit is the distance between *oriC* and *ter*); (ii) the replication time unit is defined as time required for full replication of half of the

chromosome (C period of the bacterial cell cycle); (iii) fork velocity is constant and equal to 1 time unit per length unit; (vi) the age of the genome is defined from one fork termination to the next; (v) the time at which new initiation events occur is s (the periodicity of initiation), and it is defined with respect to the previous replication initiation event; (vi) all origins activate at the same time and with the same periodicity s. Supplementary Figure 5A shows a spatiotemporal representation of the replication program for a hypothetical chromosome with two replication origins positioned at x = 0 and x = 0.5. Each new round of replication starts while the previous replication round is still ongoing, so there are four copies of newly replicated genetic material. Given the age distribution of genomes [6] (shown in Supplementary Figure 5B), the mean number of copies is calculated as an integral over all ages of age distribution multiplied by the number of copies at a particular position and a particular age (shown in Supplementary Figure 5C). Then, different compositions (percentages of genomes with one, two, or three active origins) were set as parameters. Supplementary Figure 5D shows an illustrative example with 25% genomes firing one origin and 75% firing both origins. Parameters were fitted by minimising a mean squared error (MSE) between model predicted values, F_i, and experimental data, d_i:

$$MSE = \sqrt{\frac{\sum_{i=1}^{n}(d_i - a\,F_i)^2}{n}}$$

with a scaling factor a fitted as one of the parameters, along with periodicity of initiation and percentage of genomes.

In the case of asynchronious initiation (shown in Supplementary Figure 5E), there would be differences in comparison to the synchronous initiation with a fraction of cells firing one origin. A comparison of profiles for synchronous initiation (blue curve) and asynchronous initiation (dashed magenta curve) is shown in Supplementary Figure 5F.

SUPPLEMENTARY REFERENCES

1. Bernhardt, T. G.; de Boer, P. A. J. The Escherichia coli amidase AmiC is a periplasmic septal ring component exported via the twin-arginine transport pathway. *Mol. Microbiol.* **2003**, *48*, 1171–1182.
2. Ivanova, D.; Taylor, T.; Smith, S. L.; Dimude, J. U.; Upton, A. L.; Mehrjouy, M. M.; Skovgaard, O.; Sherratt, D. J.; Retkute, R.; Rudolph, C. J. Shaping the landscape of the Escherichia coli chromosome: replication-transcription encounters in cells with an ectopic replication origin. *Nucleic Acids Res.* **2015**, *43*, 7865–7877, doi:10.1093/nar/gkv704.
3. Müller, C. A.; Hawkins, M.; Retkute, R.; Malla, S.; Wilson, R.; Blythe, M. J.; Nakato, R.; Komata, M.; Shirahige, K.; de Moura, A. P. S.; Nieduszynski, C. A. The dynamics of genome replication using deep sequencing. *Nucleic Acids Res.* **2014**, *42*, e3, doi:10.1093/nar/gkt878.
4. Skovgaard, O.; Bak, M.; Løbner-Olesen, A.; Tommerup, N. Genome-wide detection of chromosomal rearrangements, indels, and mutations in circular chromosomes by short read sequencing. *Genome Res.* **2011**, *21*, 1388–1393, doi:10.1101/gr.117416.110.
5. Retkute, R.; Nieduszynski, C. A.; de Moura, A. Mathematical modeling of genome replication. *Phys. Rev. E Stat. Nonlin. Soft Matter Phys.* **2012**, *86*, 031916.
6. Sueoka, N.; Yoshikawa, H. The chromosome of Bacillus subtilis. I. Theory of marker frequency analysis. *Genetics* **1965**, *52*, 747–757.

SUPPLEMENTARY TABLES

Supplementary Table 1: Replication profile minima established by LOESS regression of the replication profiles of *E. coli* strains with one and two replication origins

Strain background	Location of terminus-proximal LOESS minima [Mbp]	Location of *oriC*–*oriX* LOESS minima [Mbp]	Arithmetic mid points [Mbp]
MG1655	1.627	n/a	1.603
oriC^{+} *oriX*$^{+}$	1.322	3.3925	1.010; 3.330
oriC^{+} *oriX*$^{+}$ *Δtus*	0.991	3.348	1.010; 3.330
oriC^{+} *oriX*$^{+}$ *rpoB*35*	1.3175	3.373	1.010; 3.330
oriC^{+} *oriX*$^{+}$ *Δtus rpoB*35*	0.967	3.360	1.010; 3.330
ΔoriC oriX^{+} *Δtus*	0.292	n/a	0.4159
ΔoriC oriX^{+} *rpoB*35*	1.9675	n/a	0.4159
ΔoriC oriX^{+} *Δtus rpoB*35*	0.2658	n/a	0.4159
ΔoriC oriX^{+}	0.658	n/a	0.4159

Supplementary Table 2: Effect of increased *dnaA* gene dosage on the doubling times in cells with one and two ectopic replication origins

Strain background	Doubling time [min]	SD	r^2
MG1655[a]	19.6	± 1.0	0.999
oriC^{+} *oriX*$^{+}$ [a]	21.0	± 0.8	0.997
oriC^{+} *oriZ*$^{+}$ [a]	21.8	± 0.8	0.996
oriC^{+} *oriX*$^{+}$ *oriZ*$^{+}$ [a]	22.7	± 2.5	0.994
MG1655 pAU101	27.1	± 2.4	0.985
oriC^{+} *oriX*$^{+}$ pAU101	28.5	± 2.9	0.995
oriC^{+} *oriZ*$^{+}$ pAU101	29.7	± 1.7	0.992
oriC^{+} *oriX*$^{+}$ *oriZ*$^{+}$ pAU101	27.7	± 1.9	0.995

a – data for constructs without *dnaA* plasmid pAU101 as in Table 3, for comparison. For details of pAU101 see Supplementary Material and Methods.

SUPPLEMENTARY FIGURES

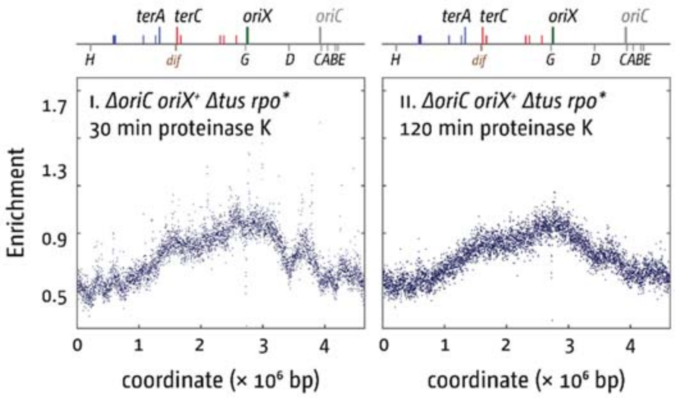

Supplementary Figure 1. Marker frequency analysis and sample quality of *E. coli ΔoriC oriX*+ *Δtus rpo** cells following short (30 min) and extended (120 min) deproteinisation via proteolytic digest using proteinase K. The numbers of reads (normalised against reads for a stationary phase wild-type control) are plotted against the chromosomal location. A schematic representation of the *E. coli* chromosome showing positions of *oriC* and *oriX* and *ter* sites (above), as well as *dif* and *rrn* operons *A–E*, *G*, and *H* (below), is shown above the plotted data. The strain used was JD1209 (*ΔoriC oriX*+ *Δtus rpo**).

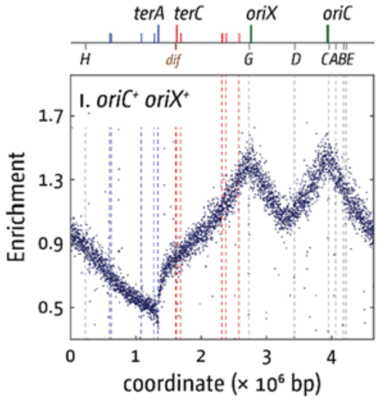

Supplementary Figure 2. Marker frequency analysis of *E. coli oriC*+ *oriX*+ cells following phenol–chloroform extraction of genomic DNA. The numbers of reads (normalised against reads for a stationary phase wild-type control) are plotted against the chromosomal location. A schematic representation of the *E. coli* chromosome showing positions of *oriC* and *oriX* (green line) and *ter* sites (above), as well as *dif* and *rrn* operons *A–E*, *G*, and *H* (below) is shown above the plotted data.

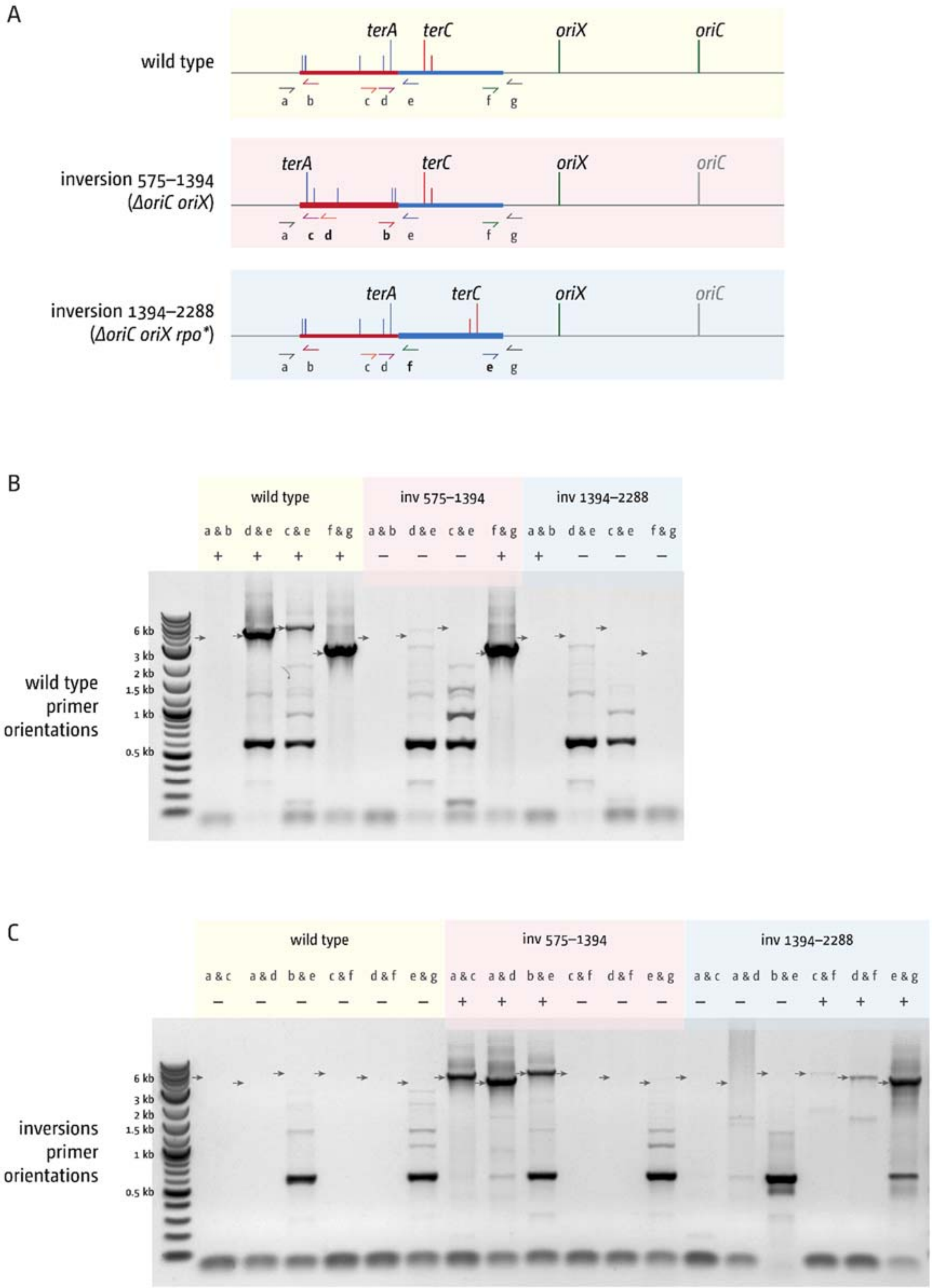

Supplementary Figure 3. PCR verification of chromosomal inversions. **A)** Schematic representation of primer binding sites, inversion locations, and the relocation of primer binding sites following specific inversion events. The schematic showing the inversion between IS5 elements at location 575 kb and 1394 kb is shaded in red, the schematic showing the inversion between IS5 elements at 1394 kb and 2288 kb is shaded blue. The wild-type

situation is shaded in yellow. Primers have a single letter identifier, which is shown in bold if the binding site is relocated due to an inversion event to highlight their changed position. Location of primer binding sites are not to scale. All expected PCR products are between 3 and 6.5 kb in length. **B)** Agarose gel electrophoresis of PCRs with primer combinations probing for the wild-type sequence and chromosomal DNA templates for a wild-type control (yellow), the *ΔoriC oriX* background carrying the inversion at IS5 elements at 575 kb and 1394 kb (red), as well as the *ΔoriC oriX rpo** background that carries an inversion at IS5 elements at 1394 kb and 2288 kb. Primer combinations as shown in A are given above each lane. The size of the PCR product for a specific primer combination is indicated by a grey arrow. The + or – indicates whether a PCR product is expected with the template used. Primer combination a and b did not give a PCR product in any PCR attempted. However, PCR products for both primers a and b are obtained if paired with different secondary primers, suggesting that it is the specific combination of a and b that fails to produce a PCR product. An inverted gel image is shown for clarity. **C)** Agarose gel electrophoresis of PCRs with primer combinations probing for both inversions and chromosomal DNA templates for a wild-type control (yellow), the *ΔoriC oriX* background carrying the inversion at IS5 elements at 575 kb and 1394 kb (red), as well as the *ΔoriC oriX rpo** background that carries an inversion at IS5 elements at 1394 kb and 2288 kb. Primer combinations as shown in A are given above each lane, with a + or – indicating whether a PCR product is expected. An inverted gel image is shown for clarity. All primers that span flanks following both inversion events show a PCR product, confirming both inversion events identified in our replication profiles.

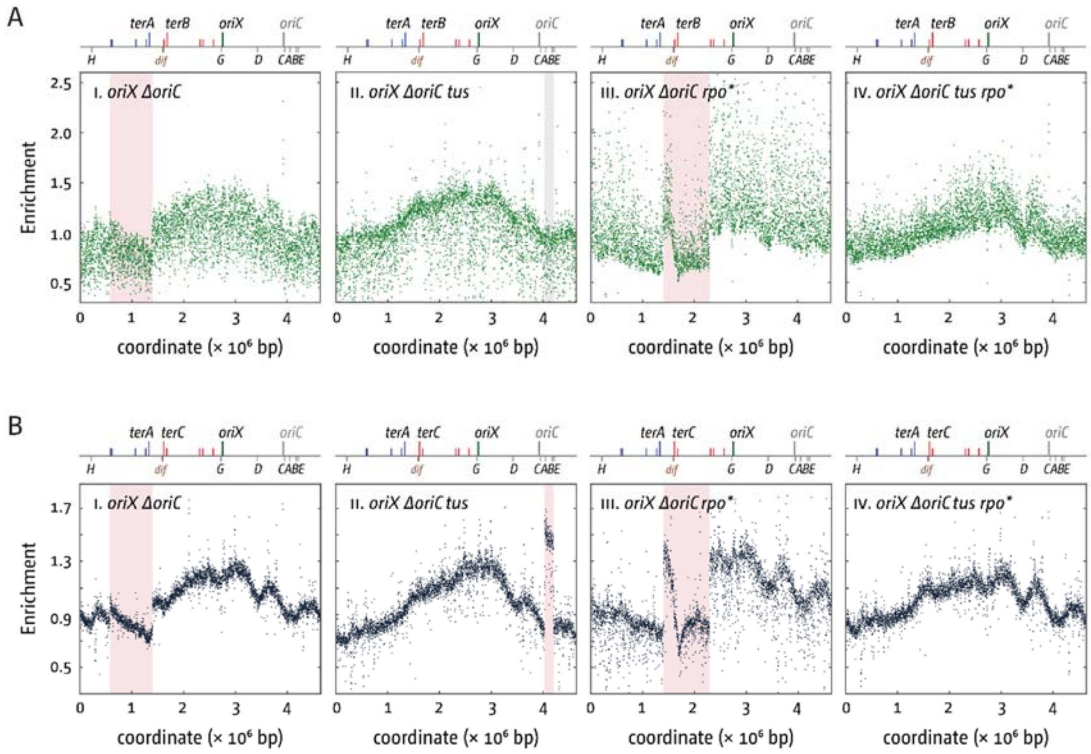

Supplementary Figure 4. Replication profiles of *E. coli* cells with synthesis starting at ectopic replication origins only. **A–B)** Marker frequency analysis of *E. coli ΔoriC oriX*+ derivatives. The numbers of reads are normalised against reads for a non-growing stationary phase wild-type control and then plotted against the chromosomal location. In this particular run, the noise observed comes from an increased overall level of noise of the entire sequencing run. This is made worse by the fact that the stationary wild-type control was particularly affected by the noise, which introduces this noise into all other samples due to the normalisation. A schematic representation of the *E. coli* chromosome showing positions of *oriC* and *oriX* (green line) and *ter* sites (above), as well as *dif* and *rrn* operons *A–E*, *G*, and *H*, is shown above the plotted data. Inverted regions are highlighted by a red box. Replication profiles in A are obtained from independent experiments, with independently generated chromosomal DNA, library generation, and sequencing runs. Replication profiles in B are reproduced from Figure 5 for comparison. The direct comparison of the *ΔoriC oriX*+ *Δtus* replication profile from the first and second run shows a duplication of the *rrnA–B* region present only in the second run, even though cultures for the preparation of genomic DNA were prepared from the same frozen stock (highlighted in red in B and in grey in A).

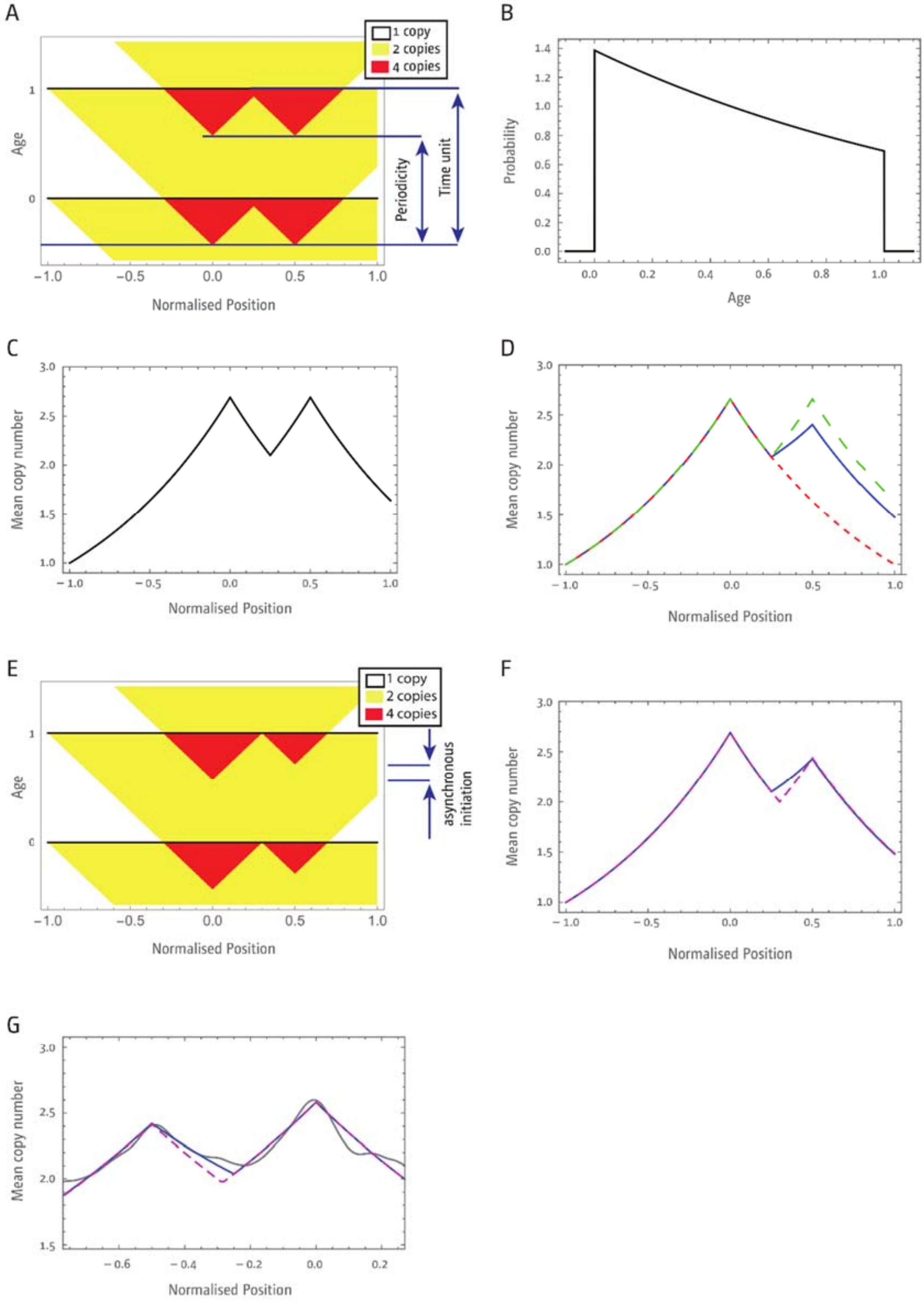

Supplementary Figure 5. Mathematical modelling of chromosomal replication in *E. coli* with one or multiple origins. **A)** Spatiotemporal representation of a replication program for two origins positioned at $x = 0$ and $x = 0.5$. The tops of each inverted red triangle indicate the initiation of replication. Number of genome copies are 1 (white), 2 (yellow), or 4 (red). The difference between two initiation events establishes the periodicity s. **B)** Age distribution. **C)**

Mean number of copies. **D)** Inferring population composition: overall profile (blue) is a result of 25% of genomes with only origin at $x = 0$ active (red) and 75% of genomes having both origins active. **E)** Spatiotemporal representation of the replication program for two asynchronously initiating origins. **F)** Mean number of copies for synchronous initiation with 25% of cells firing one origin and 75% firing two origins (blue), and asynchronous initiation with 100% of cells firing two origins but at different times (magenta). **G)** Overlay of model predictions for synchronous (blue) versus asynchronous (magenta) initiations and LOESS data of the replication profile of an *oriC*+ *oriX* strain. Asynchronous initiation predicts a shift of the termination point to the left, while a shift to the right is observed in our experimental data.
